# British Society for Rheumatology guideline on management of paediatric, adolescent and adult patients with idiopathic inflammatory myopathy

**DOI:** 10.1093/rheumatology/keac115

**Published:** 2022-03-31

**Authors:** Alexander G S Oldroyd, James B Lilleker, Tania Amin, Octavio Aragon, Katie Bechman, Verna Cuthbert, James Galloway, Patrick Gordon, William J Gregory, Harsha Gunawardena, Michael G Hanna, David Isenberg, John Jackman, Patrick D W Kiely, Polly Livermore, Pedro M Machado, Sue Maillard, Neil McHugh, Ruth Murphy, Clarissa Pilkington, Athiveeraramapandian Prabu, Phoebe Rushe, Stefan Spinty, Joanne Swan, Hasan Tahir, Sarah L Tansley, Paul Truepenny, Yvonne Truepenny, Kishore Warrier, Mark Yates, Charalampia Papadopoulou, Neil Martin, Liza McCann, Hector Chinoy, for the British Society for Rheumatology Standards, Audit and Guidelines Working Group

**Affiliations:** 1 NIHR Manchester Biomedical Research Centre, Manchester University NHS Foundation Trust, Manchester Academic Health Science Centre, Manchester, UK; 2 Centre for Musculoskeletal Research, University of Manchester, Manchester Academic Health Science Centre, Manchester, UK; 3 Centre for Epidemiology Versus Arthritis, University of Manchester, Manchester, UK; 4 Department of Rheumatology, Salford Royal NHS Foundation Trust, Salford, UK; 5 Manchester Centre for Clinical Neurosciences, Salford Royal NHS Foundation Trust, Manchester Academic Health Science Centre, Salford, UK; 6 Department of Paediatric Rheumatology, Leeds Children’s Hospital, Leeds, UK; 7 Pharmacy Department, Alder Hey Children’s Hospital NHS Foundation Trust, Liverpool, UK; 8 School of Pharmacy and Biomolecular Sciences, Liverpool John Moores University, Liverpool, UK; 9 Centre for Rheumatic Diseases, King’s College London, London, UK; 10 Department of Paediatric Rheumatology, Royal Manchester Children’s Hospital, Manchester, UK; 11 Department of Rheumatology, King’s College Hospital NHS Foundation Trust, London, UK; 12 Department of Health Professions, Manchester Metropolitan University, Manchester, UK; 13 Department of Rheumatology, North Bristol NHS Trust, Bristol, UK; 14 Department of Clinical and Academic Rheumatology, University of Bristol, Bristol, UK; 15 Queen Square Centre for Neuromuscular Diseases, UCL Queen Square Institute of Neurology, University College London, London, UK; 16 Department of Rheumatology, Division of Medicine, University College London, London, UK; 17 Department of Rheumatology, Nuffield Orthopaedic Centre, Oxford, UK; 18 Department of Rheumatology, St George’s University Hospitals NHS Foundation Trust, London, UK; 19 Institute of Medical and Biomedical Education, St George’s, University of London, London, UK; 20 Department of Paediatric Rheumatology, Great Ormond Street Hospital NHS Foundation Trust, London, UK; 21 NIHR Great Ormond Street and University College London Biomedical Research Centre, London, UK; 22 Department of Neuromuscular Diseases, Centre for Rheumatology, University College London, London, UK; 23 NIHR University College London Hospitals Biomedical Research Centre, University College London Hospitals (UCLH) NHS Foundation Trust, London, UK; 24 Department of Rheumatology, Northwick Park Hospital, London North West University Healthcare NHS Trust, London, UK; 25 Department of Pharmacy and Pharmacology, University of Bath, Bath, UK; 26 Department of Dermatology, Sheffield University Teaching Hospitals, Sheffield, UK; 27 Rheumatology Research Group, Institute of Inflammation and Aging, University of Birmingham, Birmingham, UK; 28 Department of Rheumatology, Sandwell and West Birmingham NHS Foundation Trust, Birmingham, UK; 29 Patient Representative; 30 Department of Paediatric Neurology, Alder Hey Children's NHS Foundation Trust, Liverpool, UK; 31 Juvenile Dermatomyositis Parent Representative; 32 Department of Rheumatology, Royal Free London NHS Trust, London, UK; 33 Division of Medicine, University College London, London, UK; 34 Royal National Hospital for Rheumatic Diseases, Royal United Hospitals Bath NHS Foundation Trust, Bath, UK; 35 Relative/Caregiver; 36 Department of Paediatric Rheumatology, Nottingham Children’s Hospital, Nottingham University Hospitals NHS Trust, Nottingham, UK; 37 Department of Paediatric Rheumatology, Royal Hospital for Children, Glasgow, UK; 38 Scottish Paediatric & Adolescent Rheumatology Network, Glasgow, Scotland; 39 Department of Paediatric Rheumatology, Alder Hey Children’s NHS Foundation Trust, Liverpool, UK

**Keywords:** myositis, muscle, adolescent rheumatology, paediatric/juvenile rheumatology, DMARDs, immunosuppressants


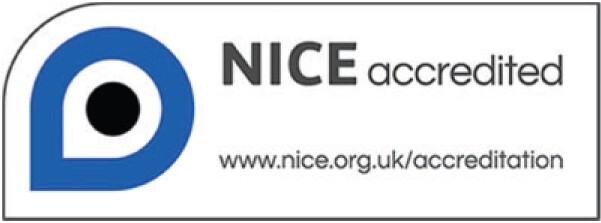


NICE has accredited the process used by BSR to create its clinical guidelines. The term began on 27 February 2012 and the current renewed accreditation is valid until 28 January 2023. More information on accreditation can be viewed at www.nice.org.uk/accreditation.

## Scope and purpose

### Background

Idiopathic inflammatory myopathy (IIM) is a multi-system autoimmune condition characterised by muscle inflammation (myositis), interstitial lung disease (ILD), and skin manifestations with an incidence of up to 19 per 1,000,000 person-years in adults and up to 4 per 1,000,000 person-years in children. Estimated UK prevalence (for adult-onset IIM) is 10, 000 [1, 2].

### Need for guideline

No rigorously produced evidence-based guidelines for IIM spanning juvenile and adult-onset disease exists. Assimilating key research relating to management and formation of practical evidence-based recommendations will aid clinicians and help optimize management and outcomes.

### Target audience

The target readership is clinicians caring for patients with IIM, including paediatric and adult rheumatologists, neurologists, dermatologists, respiratory physicians, oncologists, gastroenterologists, and cardiologists. Rheumatology and neurology nurses, physiotherapists, occupational therapists, podiatrists, speech and language therapists, specialist rheumatology pharmacists, and psychologists will also find these recommendations relevant.

### Areas the guideline does not cover

Diagnosis, classification, and investigation of suspected IIM are not addressed. Inclusion body myositis is not covered.

### Stakeholder involvement

The project was led by an executive committee (J.B.L., A.G.S.O., H.C., N.M., L.M.). A multidisciplinary working group was convened with input from rheumatologists (D.I., H.G., H.T., N.Mc., A.P., P.G., S.T., H.C., P.K., P.M.M., A.G.S.O.), paediatric rheumatologists (C.Pi., C.Pa., N.Ma., K.W., L.M., T.A.), neurologists (J.B.L., M.H.), a paediatric neurologist (S.Sp.), a nurse (P.L.), a pharmacist (O.A.), a dermatologist (S.M.), paediatric dermatologist (R.M.), physiotherapists (V.C., S.M., W.J.G.), and a former GP/Specialty Doctor in Rheumatology (J.J.). Lay (patient and relative) input was also received throughout the process (P.T., Y.T., J.S., P.R.). The guideline production process was informed by a EULAR Recommended Methodologist (P.M.M.) and literature searches were carried out by experts at the Centre for Rheumatic Diseases, Kings College London (J.G., K.B., M.Y.).

### Rigour of development

This guideline was developed in line with the BSR Creating Clinical Guidelines Protocol using AGREEII (Appraisal of Guidelines for Research and Evaluation II) methodology.

### Selection of key questions

Starting March 2018, the executive committee and working group agreed the guideline scope and created key questions structured using the PICO (patient or population, intervention, comparison, outcome) format. Each question was subdivided into focused clinical questions during the evidence review and recommendation formulation process.

### Literature search—scope and search strategy

Using key questions as a basis, a literature search was undertaken using Ovid (see ‘Search terms’ in Supplementary Material S1, available at *Rheumatology* online). Search results and additional manually identified references up to October 2020 were included. Evidence published after October 2020 was not included as this was the cut-off for eligibility. A potential limitation of this guideline is that relevant literature may have been published since October 2020; data or information from these studies could not be included in the recommendation formation process.

### Eligibility criteria

Published peer reviewed clinical studies relating to any IIM subtype except inclusion body myositis were included. Case reports/series were limited to those describing outcomes for three or more subjects. Review articles, editorials, conference proceedings, and existing clinical guidelines were excluded. Non-English language papers were excluded unless a translation was published. Basic science studies without clear clinical applicability were excluded. Abstracts of papers were reviewed by two authors to determine eligibility against these criteria (PRISMA flow diagram shown in Supplementary Fig. S1, available at *Rheumatology* online).

### Methods used to formulate recommendations

The full text of each eligible paper was reviewed by two assessors using Grading of Recommendations, Assessment, Development, and Evaluations (GRADE) methodology. Each reference was categorized as high (A), moderate (B) or low/very low (C) quality. A third assessor resolved disagreements.

A total of 213 papers were used to form recommendations. See Supplementary Table S1 (available at *Rheumatology* online) for details of evidence base contributing to recommendations.

Draft recommendations were created and categorized as applicable to all patients, adult-specific or paediatric-specific. The process outlined by the Scottish Intercollegiate Guidelines Network (SIGN) [3] was used to summarize the quality of body of evidence for each recommendation: high (A), moderate (B), low (C) or very low (D), according to GRADE methodology.

Content, wording, strength of recommendation (strong = 1, conditional = 2), and quality of supporting evidence for each recommendation were subjected to a formal consensus building process using a combination of face-to-face meetings and online surveys. Strength of agreement (SoA) for finalized recommendations was determined using a simple binary voting system for each voter and is presented as a percentage. Authors were free to abstain from voting on areas where they did not feel clinically competent, with the percentage reflecting voters. Only recommendations with a SoA >80% were included in the guideline.

### Policy for updates

Requirement for updates will be considered by the BSR Standards, Audit, and Guidelines Working Group and according to principles outlined in the BSR Creating Clinical Guidelines Protocol.

## Recommendations

Recommendations are followed by parentheses detailing GRADE and SoA details (strength of recommendation, quality of body of evidence, SoA).

### (i) How should skeletal muscle inflammation (myositis) be treated?

**1**-High dose glucocorticoids should be used to treat active muscle inflammation at time of treatment induction (1, B, 100%).

**1a**-**Adult-specific**. Oral prednisolone at a dose of 0.5–1 mg/kg/day, usually 40–60 mg, is recommended (1, B, 100%).

**1b**-**Paediatric-specific**. Oral prednisolone at a dose of 1–2 mg/kg/day or intravenous methylprednisolone pulses 30 mg/kg/day, maximum 1 g daily i.v. dose is recommended (1, B, 100%).

**1c**-Intravenous methylprednisolone is to be considered, especially when there are concerns about gastrointestinal absorption. Use of intravenous methylprednisolone may allow increased therapeutic effect and less toxicity compared with oral glucocorticoid (2, B, 96%).

**2**-Oral prednisolone should be tapered according to clinical response (1, B, 100%).

**3**-Disease modifying anti-rheumatic drugs should be used to reduce muscle inflammation, achieve clinical remission and reduce steroid burden (1, C, 100%).

**3a**-**Paediatric-specific**. Early, complete control of muscle weakness and inflammation should be sought in juvenile-onset IIM, with the aim of improving outcomes and reducing disease-related complications (1, B, 100%).

**3b**-**Paediatric-specific**. A combination of high dose glucocorticoid and methotrexate should be used as first-line treatment in most cases (1, B, 100%).

**3c**-**Paediatric-specific**. A combination of prednisolone and methotrexate, as opposed to prednisolone and ciclosporin, should be used for the treatment of juvenile-onset IIM as this has a more favourable side effect profile (1, B, 100%).

**3d**-**Paediatric-specific**. Mycophenolate mofetil is to be considered as a treatment option to improve skin and muscle disease (2, C, 100%).

**3e**-**Adult-specific**. Methotrexate, azathioprine, tacrolimus, ciclosporin, and mycophenolate mofetil are to be considered for the treatment of active myositis and long-term maintenance of disease remission (2, C, 96%).

**4**-Intravenous immunoglobulin should be considered as a treatment of severe and/or refractory muscle inflammation (1, B, 100%).

**5**-Management of IIM should include a safe and appropriate exercise programme led and monitored by a specialist physiotherapist and/or a specialist occupational therapist to improve quality of life and function (1, B, 100%).

**6**-Rituximab is to be considered as a treatment option for refractory myositis and may be particularly effective in (2, A, 100%):

- Juvenile-onset disease
- Patients with a positive myositis autoantibody profile
- Patients with lower burden of disease damage

**7**-Cyclophosphamide should be considered as a treatment option for severe and/or refractory IIM (1, B, 100%).

**8**-**Adult-specific**. Abatacept is to be considered as a treatment option in refractory adult IIM (2, B, 100%).

Glucocorticoids are crucial for myositis remission induction and maintenance. Glucocorticoid dose should be weaned when disease activity, considered across all domains, substantially improves, usually after around 6 weeks of treatment initiation. Available evidence precludes evidence-based recommendations regarding rate of glucocorticoid dose reduction. Whilst dosages per kilogram are included in recommendations for juvenile onset disease, it is important to note that ceiling doses may apply. Steroid-free remission can be facilitated using DMARDs and/or additional immunosuppressive/immunomodulatory treatments. Evidence exists to support use of conventional synthetic DMARDs (csDMARDs) (tacrolimus, azathioprine, methotrexate, ciclosporin, mycophenolate mofetil) alongside glucocorticoids early in the disease course to induce and maintain remission, although conflicting results exist in some cases [4–10]. Evidence does not exist to allow recommendation of specific csDMARDs as first-/second-/third-line for adults. DMARDs should be prescribed and monitored according to existing age-appropriate BSR guidelines [11, 12].

Exercise is safe and effective for people with IIM and can improve quality of life and function. Specialist physiotherapy and occupational therapy input is important for management of patients with IIM and should be considered in service planning to ensure appropriate access for all patients.

Evidence exists allowing recommendation of use of ‘second-line’ treatments, such as CYC, rituximab (RTX), IVIG and abatacept, for patients with persistent active disease despite glucocorticoid and csDMARD therapy. A prospective, double-blind, randomized, placebo-controlled phase III study, completed after the cut-off date for evidence inclusion, has demonstrated efficacy of IVIG [13].

CYC is an option for severe and/or refractory IIM. Route of administration should be considered since intravenous (i.v.) CYC (intermittent pulses), compared with oral CYC, is associated with fewer side effects. CYC is usually administered by i.v. infusion [14], reducing risk of leucopenia, haemorrhagic cystitis, and gonadal toxicity [15, 16].

RTX and IVIG are options for management of active IIM (e.g. myositis, dysphagia, refractory skin disease) refractory to glucocorticoid/csDMARD-based immunosuppression. In England, RTX and IVIG can only be used according to NHS England (NHSE) commissioning stipulations and should be prescribed in conjunction with a specialist centre [17, 18]. NHSE guidance does not apply in Wales, Northern Ireland or Scotland. In Scotland, the National Plasma Products Expert Advisory Group (NPPEAG) indicates IVIG as appropriate for patients with resistant or aggressive disease [19]. A single prospective delayed-start study has demonstrated the benefit of abatacept in adult-onset IIM [20]. Future studies are required to confirm efficacy.

At time of recommendation consensus forming there was insufficient evidence to recommend anti-TNF-α therapy for treatment of myositis. There was also insufficient evidence to recommend use of Janus kinase (JAK) inhibitors in IIM treatment; however, published case series are promising and future clinical trials may provide a stronger evidence base [21–23].

### (ii) How should IIM-related skin manifestations be treated?

**1**-Rituximab is to be considered for the treatment of skin disease refractory to glucocorticoid/csDMARD-based immunosuppression (2, B, 100%).

**2**-IVIG should be considered for the treatment of skin disease refractory to glucocorticoid/csDMARD-based immunosuppression (1, B, 100%).

**3**-Sun avoidance and regular use of high factor broad spectrum sun cream is to be considered to reduce likelihood of a disease flare affecting skin or muscle (2, C, 100%).

**4**-**Paediatric-specific**. Systemic immunosuppressive drugs are to be considered for the treatment of ongoing skin disease activity, including reduced nailfold capillary density (2, C, 100%).

**5**-**Paediatric-specific**. An early increase in treatment is to be considered in patients with persistent skin disease to aid remission and reduce development of calcinosis (2, C, 100%).

Inadequate evidence exists to allow recommendation of topical agents to treat IIM-specific skin manifestations; however, topical tacrolimus and glucocorticoids could be considered alongside dermatology input.

Evidence relating to treatment of IIM-related skin manifestations is limited; however, studies indicate the ability of both IVIG and RTX to treat skin manifestations refractory to glucocorticoid/csDMARD-based immunosuppression. Nailfold capillary abnormalities in children with IIM can reflect systemic disease activity and should be considered when making treatment decisions [24].

Studies indicate sun exposure is associated with cutaneous and non-cutaneous DM and JDM disease flares [25]. Sun avoidance may thus form part of the management strategy for DM/JDM.

### (iii) How should IIM-related ILD be managed?

**1**-**Paediatric-specific**. Routine assessment of pulmonary function, including measurement of diffusing capacity or transfer factor of the lung for carbon monoxide (DLCO or TLCO) in juvenile-onset IIM should be performed, as pulmonary function abnormalities are frequent and may be asymptomatic (1, B, 100%).

**2**-**Adult-specific**. Interstitial lung disease should be screened for in high-risk patients (1, B, 100%).

**3**-**Adult-specific**. In the treatment of rapidly progressive interstitial lung disease (RP-ILD):

- Induction therapy with high dose steroids is to be considered (2, C, 96%).
- The use of ciclosporin or tacrolimus, alongside steroids, is to be considered in patients with RP-ILD (2, C, 96%).
- Cyclophosphamide or rituximab therapy is to be considered early, potentially as part of the induction regimen (2, C, 96%).

**4**-**Adult-specific**. In the treatment of chronic IIM-associated interstitial lung disease:

- Immunosuppression using steroids with or without a single DMARD (azathioprine, ciclosporin, tacrolimus, mycophenolate) is to be considered (2, C, 100%).
- Rituximab or cyclophosphamide is to be considered in treatment-resistant patients (2, C, 100%).

IIM-related ILD management should be carried out alongside ILD-specialist respiratory physicians. ILD risk is increased with anti-synthetase syndrome, presence of an anti-synthetase-associated autoantibody, anti-melanoma differentiation-associated protein 5 autoantibody positivity, and scleroderma overlap. ILD screening methods include plain chest X-ray radiography, pulmonary function tests (including DLCO), and where indicated, high resolution CT scanning. Insufficient evidence exists to advise ILD screening frequency.

Insufficient evidence exists to form recommendations regarding pharmacological management of IIM-associated ILD in paediatric patients.

### (iv) What management steps should be taken to reduce fracture risk in people with IIM?

**1**-**Adult-specific**. A bone health assessment should be performed, regardless of glucocorticoid therapy, and appropriate management instigated (1, B, 100%).

Fracture risk consideration in IIM is important given glucocorticoid use, female preponderance, and average age of onset for adult disease [26]. Fragility fracture risk assessment should be carried out in accordance with NICE guidance at time of diagnosis and whenever risk factors change [27]. Glucocorticoid weaning, once remission is attained, may reduce fragility fracture risk.

Studies, although limited by small populations, suggest JDM is associated with increased vertebral fracture risk, even before substantial corticosteroid exposure [28].

### (v) What key prognostic and management factors should be considered for children with IIM?

**1**-**Paediatric-specific**. Juvenile-onset IIM should be managed by paediatric specialists as it differs from adult-onset IIM in several ways, including greater presence of subcutaneous calcification, less disease damage, lack of association with cancer, increased risk of vasculitis, and different autoantibody associations (1, C, 95%).

**2**-**Paediatric-specific**. Shorter time to diagnosis is associated with improved disease outcome, therefore early referral to a specialist service is to be considered (2, C, 100%).

**3**-**Paediatric-specific**. Age-specific considerations should be taken into account when using tools that measure muscle strength, function, and quality of life (1, B, 100%).

**4**-**Paediatric-specific**. Healthcare professionals should look for signs of connective tissue disease overlap, which is associated with increased risk of mortality (1, C, 89%).

**5**-**Paediatric-specific**. Patients with juvenile-onset IIM should be assessed for calcinosis (1, C, 100%).

Age appropriate tools such as the Childhood Myositis Assessment Score, Childhood Health Assessment Questionnaire, and Juvenile Dermatomyositis Multidimensional Assessment Report should be used to assess muscle strength, function, and quality of life [29, 30]. There is significantly higher mortality in patients with overlapping connective tissue disease features compared with those with JDM [31]. Patients should therefore be carefully screened for overlapping connective tissue disease features and wider organ involvement.

Factors associated with increased risk of calcinosis include younger age at disease onset, particularly disease onset in infancy, delay to diagnosis or delay to treatment initiation, more severe disease, prolonged disease duration, and presence of anti-nuclear matrix protein 2 (NXP2) autoantibodies [32, 33]. Clinical examination and plain X-ray radiography can be used to identify calcinosis.

### (vi) Is autoantibody testing useful in people with IIM?

**1**-Patients should be tested for myositis auto-antibodies (1, B, 100%).

Myositis-specific antibodies and myositis-associated autoantibodies can facilitate diagnosis, inform disease phenotype and prognosis, and may help tailor treatment [34, 35]. Interpretation of immunoblot results should be carried out in the context of the patient’s overall clinical presentation. Autoantibody titres should not be used to monitor disease activity.

### (vii) How should cancer be screened for in people with an IIM?

**1**-**Paediatric-specific**. Routine screening for cancer is not warranted in juvenile-onset IIM (1, B, 100%).

**2**-**Adult-specific**. The risk of cancer should be considered in all patients and screening should be particularly considered in those with the following risk factors (1, B, 100%):

- Older age at onset
- Male gender
- Dysphagia
- Cutaneous necrosis
- Resistance to immunosuppressive therapy
- Rapid disease onset
- Positive anti-TIF1-γ autoantibodies
- Positive anti-NXP2 autoantibodies
- Negative for known myositis-specific autoantibodies

There is an association between adult-onset IIM and malignancy. Evidence pertaining to effective cancer screening is limited but indicates the utility of CT scanning of the thorax, abdomen, and pelvis for at-risk patients, such as anti-transcriptional intermediary factor-1γ (anti-TIF1-γ) positive patients. Tumour markers and ^18^F-FDG PET/CT scanning can be considered in selected patients.

In contrast with adult-onset IIM, juvenile onset IIM is not associated with cancer, with literature consisting only of isolated case reports. Routine cancer screening in juvenile-onset IIM is not advised unless underlying cancer is suspected.

### (viii) How should IIM treatment during pregnancy and the breastfeeding period be amended?

**1**-Those wishing to conceive should be advised to plan conception whilst their disease is well controlled (1, B, 100%).

**2**-Pregnancy should be managed in conjunction with maternal medicine specialists (1, B, 96%).

**3**-Increased vigilance is required post-partum as patients may be at risk of disease flare (1, C, 96%).

Pregnancy should be managed alongside maternal medicine specialists due to lower mean birth weight, increased risk of obstetric complications, such as pre-eclampsia and eclampsia, and longer hospitalization duration during delivery. Evidence, although limited, indicates good IIM control is associated with better pregnancy outcomes [36]. Conception should be planned once disease remission is established using medications compatible with pregnancy according to the BSR guideline on prescribing drugs in pregnancy and breastfeeding [37].

### (ix) How should IIM-related cardiovascular disease be assessed for and treated?

**1**-**Adult-specific**. Patients should undergo a regular cardiovascular risk assessment (1, C, 100%).

**2**-**Paediatric-specific**. Assessment and management of cardiovascular risk factors is to be considered, including hypertension, obesity or metabolic abnormalities (lipids/insulin resistance) (2, C, 100%).

IIM is associated with an increased incidence of hypertension, diabetes, dyslipidaemia, obesity, and coronary artery disease (adult-specific) representing an opportunity for intervention to reduce cardiovascular risk [38, 39]; however, insufficient evidence exists to advise screening frequency.

Micro-vasculopathy and glucocorticoid treatment are considered responsible for the hypertension observed in 25–50% of patients with JDM [40]. Studies have identified altered cardiovascular risk factors in JDM patients [41] that may lead to increased risk of early atherosclerosis later in adulthood [42].

### (x) How should cardiac involvement in IIM be screened for?

**1**-**Adult-specific**. Patients should undergo screening for cardiac involvement; serum cardiac damage markers, ECG, echocardiography, and cardiac MRI are to be considered (2, B, 100%).

**2**-**Adult-specific**. Cardiac troponin I (not cardiac troponin T) should be used as the preferred serum marker for screening and monitoring cardiac involvement (1, B, 100%).

**3**-**Paediatric-specific**. Screening for cardiac involvement in patients with juvenile-onset IIM with ECG and echocardiogram is to be considered (2, C, 100%).

Cardiac myositis is associated with increased morbidity and mortality risk. Raised serum cardiac damage markers may indicate cardiac involvement. However, some ‘cardiac-specific’ markers, particularly cardiac troponin T, can also be expressed and released from regenerating skeletal muscle, potentially causing ambiguity. Measuring cardiac troponin I is recommended. Several cardiac abnormalities, including left ventricular dysfunction, ECG abnormalities, and reduced heart rate variability [43, 44], have been reported in people with IIM.

### (xi) How should IIM-related dysphagia be screened for and managed?

**1**-Routine assessment of dysphagia is to be considered in all patients (2, C, 92%).

**2**-Swallowing assessment and involvement of speech and language therapist/gastroenterology teams is to be considered in those with dysphagia (2, C, 100%).

**3**-IVIG therapy for active disease and dysphagia resistant to other treatments is to be considered (2, C, 100%).

Dysphagia is common, impacts upon quality of life, and is associated with weight loss and aspiration pneumonia, which can be fatal. Swallowing dysfunction may not always be predicted by generalized muscle weakness [45]. Risk is increased with anti-NXP2 positivity or malignancy [46]. Clinicians should routinely enquire for dysphagia-related symptoms and consider early involvement of speech and language therapists when required.

Dysphagia is recognized as an indication for IVIG treatment by NHSE [18]. IVIG and other immunomodulatory therapies including glucocorticoid, csDMARDs (methotrexate, azathioprine, ciclosporin, tacrolimus, mycophenolate mofetil, hydroxychloroquine), CYC, and RTX have been reported to improve symptoms of dysphagia and/or objective swallow assessments.

### (xii) How should quality of life and mental wellbeing be assessed and treated in people with IIM?

**1**-Psychological wellbeing and psychiatric comorbidities should be assessed (1, C, 92%).

**2**-Psychological wellbeing and health-related quality of life should be routinely assessed using an age-appropriate tool (1, B, 100%).

**3**-Factors negatively impacting upon health-related quality of life (e.g. skin involvement, pruritis, steroid adverse effects) should be addressed (1, C, 96%).

**4**-**Paediatric-specific**. Factors negatively impacting upon health-related quality of life in children include pain, muscle weakness, and poor sleep, and should be managed appropriately (1, C, 95%).

**5**-Individually tailored exercise and/or rehabilitation should be encouraged across all ranges of disease activity with the aim of improving psychological wellbeing (1, B, 96%).

**6**-Where relevant, targeted exercises given by a specialist physiotherapist and/or a specialist occupational therapist to improve grip strength should be considered, due to the negative impact of poor grip strength on activities of daily living and quality of life (2, C, 96%).

Significant deficits are evident in measures of health-related quality of life (HRQoL) in both adult and juvenile-onset IIM. Evidence suggests a number of IIM-specific factors that can negatively impact HRQoL, such as active disease, increased functional impairment, and decreased muscle strength [47]. HRQoL can be assessed using tools such as the Child Health Questionnaire (CHQ-50) and the 36-Item Short Form Survey (adult-specific) [48]. Minimizing functional impairment via specialist physiotherapy and/or occupational therapy should be considered. Screening for concerns such as low mood and anxiety, and offering psychological interventions as early possible where needed can be considered.

### (xiii) What IIM management considerations should be made for certain ethnic groups?

**1**-Ethnicity is to be considered when assessing patients; clinical manifestations, associated autoantibodies, and underlying risk factors may vary according to ethnicity (2, C, 96%).

Ethnic minority groups appear to be at increased risk of anti-signal recognition particle (anti-SRP) autoantibody-related disease, increased cardiovascular risk, and more at risk of juvenile polymyositis/juvenile connective tissue myopathy. Calcinosis rates are higher in black children with JDM in North American [49] and South African cohorts [50].

### Applicability and utility

Supplementary Fig. S2 (available at *Rheumatology* online) shows an overview of recommendations. There should be no barriers to implementation in the UK. Use of the audit tool (Supplementary Table S2, available at *Rheumatology* online) is encouraged.

This guideline highlights the limited high-quality evidence base available for IIM, with relative absence of RCTs or head-to-head comparison of treatments. Recommendations are therefore predominantly based on observational studies. Controlled trials are crucial to further evaluate promising treatments. Long-term outcomes especially related to cardiovascular or cerebrovascular risks needs better definition. Impact of IIM on mental health and quality of life should not be underestimated. Patients and carers should be fully integrated in defining priorities for future IIM research.

## Supplementary Material

keac115_Supplementary_Data
