# Supplementary material for: British Society for Rheumatology guideline on management of paediatric, adolescent and adult patients with idiopathic inflammatory myopathy
Source: Rheumatology (Oxford). 2022 Mar 31;61(5):1760–8. doi: 10.1093/rheumatology/keac115 (PMC9398208; doi:10.1093/rheumatology/keac115)
Supplement: keac115_Supplementary_Data [file keac115_supplementary_data.zip › keac115-suppl_data/Suppl_fig_S1_-_PRISMA_2020_flow_diagram_-_Final_FL.docx]

**Supplementary Figure S1 - PRISMA flow diagram of included/excluded studies**

**Identification of studies via databases and registers**

Records removed *before screening*:

Duplicate records removed

(n = 1113)

Records identified from:

Databases (n = 6091)

**Identification**

Records screened

(n = 4978)

Records excluded

(n = 4585)

**Screening**

Reports excluded:

Ineligible (n = 180)

Reports assessed for eligibility

(n = 393)

Studies included

(n = 213)

**Included**
