# Supplementary material for: British Society for Rheumatology guideline on management of paediatric, adolescent and adult patients with idiopathic inflammatory myopathy
Source: Rheumatology (Oxford). 2022 Mar 31;61(5):1760–8. doi: 10.1093/rheumatology/keac115 (PMC9398208; doi:10.1093/rheumatology/keac115)
Supplement: keac115_Supplementary_Data [file keac115_supplementary_data.zip › keac115-suppl_data/Suppl_material_S1_-_Literature_search_terms_-_Final_FL (1).docx]

Supplementary Material S1

Question 1.

How do we manage and treat different patterns (e.g. monophasic, chronic progressive, relapsing remitting) and organ specific disease in idiopathic inflammatory myopathy (IIM)?

“Idiopathic inflammatory myopathy” or “Polymyositis” or “Dermatomyositis” or “Immune mediated necrotising myopathy” or “Antisynthetase syndrome” or “Amyopathic dermatomyositis” or “Juvenile dermatomyositis” or “Connective tissue disease overlap myositis” or “Juvenile myositis”

AND

"prednisolone" or "corticosteroid*" or "adrenocorticotropic*" or "ACTH" or "azathioprine" or "methotrexate" or "ciclosporin" or "tacrolimus" or "calcineurin*" or "mycophenolate" or "chlorambucil" or "cyclophosphamide" or "IVIg" or "immunoglobulin" or "interferon" or "plasma exchange" or "fingolimod" or "ajulemic acid" or "siponimod" or "rituximab" OR "belimumab" OR "infliximab" OR "etanercept" OR "tocilizumab" OR "abatacept" OR "adalimumab" OR "interferon alpha blockers" OR "anakinra" (see notes section at end of document)

AND

"IMACS" or "PRINTO" or "muscle strength" or "MMT8" or "MMT26" or "global activity" or "physical function" or "HAQ" or "CHAQ" or "CMAS" or "muscle enzyme" or "MDAAT" or "disease activity score" or "myositis damage index”


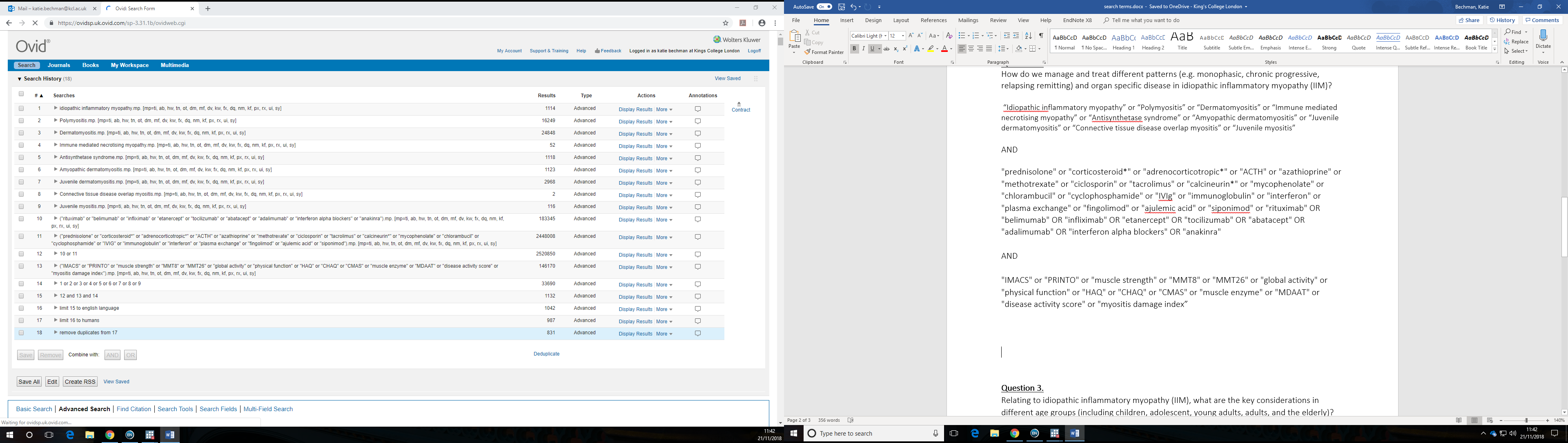


Question 2.
Relating to idiopathic inflammatory myopathy (IIM), what are the key considerations in different age groups (including children, adolescent, young adults, adults, and the elderly)?

“Idiopathic inflammatory myopathy” or “Polymyositis” or “Dermatomyositis” or “Immune mediated necrotising myopathy” or “Antisynthetase syndrome” or “Amyopathic dermatomyositis” or “Juvenile dermatomyositis” or “Connective tissue disease overlap myositis” or “Juvenile myositis”

AND

"Age" or "Children" or "Adolescent" or "Young adult" or "Adult" or "elderly"

AND

"IMACS" or "PRINTO" or "muscle strength" or "MMT8" or "MMT26" or "global activity" or "physical function" or "HAQ" or "CHAQ" or "CMAS" or "muscle enzyme" or "MDAAT" or "disease activity score" or "myositis damage index" or "Pulmonary function testing" or "Cutaneous dermatomyositis activity and severity index" or "MRI" or "Work disability and work productivity" or "serious adverse events" or "mortality"


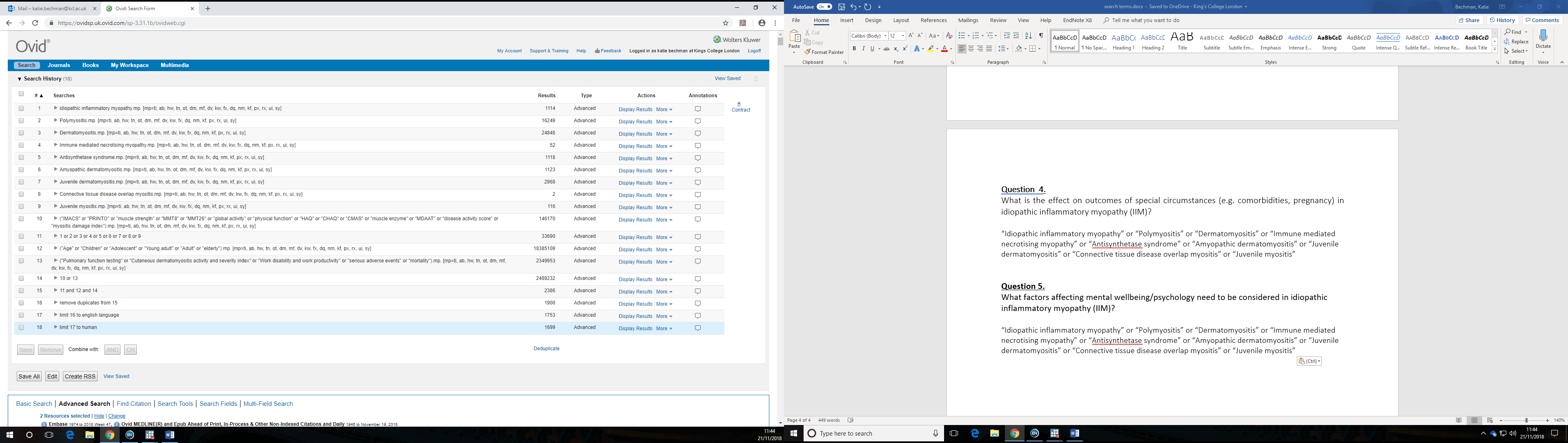


Question 3.

What is the effect on outcomes of special circumstances (e.g. comorbidities, pregnancy) in idiopathic inflammatory myopathy (IIM)?

“Idiopathic inflammatory myopathy” or “Polymyositis” or “Dermatomyositis” or “Immune mediated necrotising myopathy” or “Antisynthetase syndrome” or “Amyopathic dermatomyositis” or “Juvenile dermatomyositis” or “Connective tissue disease overlap myositis” or “Juvenile myositis”

AND

“pregnan*” or “comorbidit*” or “obesity” or “diabetes” or “smoking”


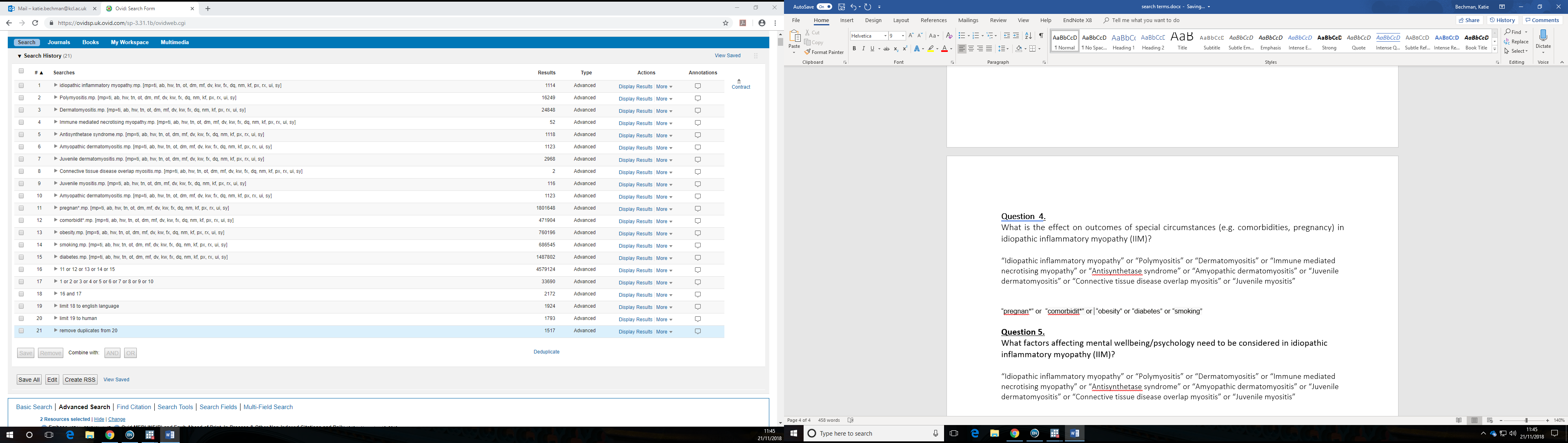


Question 4.

What factors affecting mental wellbeing/psychology need to be considered in idiopathic inflammatory myopathy (IIM)?

“Idiopathic inflammatory myopathy” or “Polymyositis” or “Dermatomyositis” or “Immune mediated necrotising myopathy” or “Antisynthetase syndrome” or “Amyopathic dermatomyositis” or “Juvenile dermatomyositis” or “Connective tissue disease overlap myositis” or “Juvenile myositis”

AND

“Mental health” or “Psych*” or “Anxiety” or “Quality of life” or “Socio” or “Depress*”


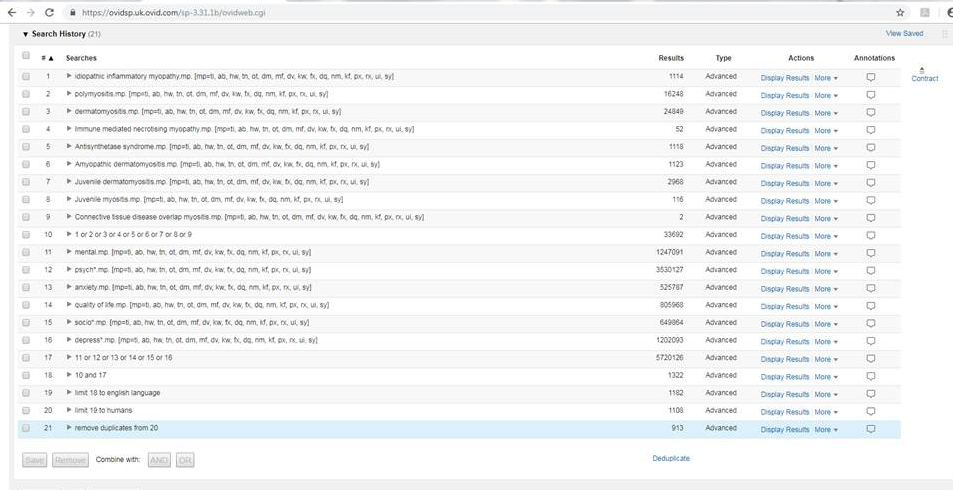


**Notes**

- The literature search was initially performed in October 2018
- It was repeated on 21/6/19 with the following changes:
  - JAK inhibitors were added to PICO2 (tofacitinib, baricitinib, upadacitinib and Filgotinib)
