# Supplementary material for: British Society for Rheumatology guideline on management of paediatric, adolescent and adult patients with idiopathic inflammatory myopathy
Source: Rheumatology (Oxford). 2022 Mar 31;61(5):1760–8. doi: 10.1093/rheumatology/keac115 (PMC9398208; doi:10.1093/rheumatology/keac115)
Supplement: keac115_Supplementary_Data [file keac115_supplementary_data.zip › keac115-suppl_data/Suppl_table_S1_-_Recommendations_and_corresponding_evidence_-_Final_FL.docx]

**Supplementary Table S1 - Individual recommendations and corresponding evidence**

| **Number** | | **Statement (unless otherwise specified, recommendation applies to adults and children)** | | | **Cited evidence** | **Strength/**  **Quality/**  **Agreement** | | | | **Number of raters**  **Numerator/denominator** | | |  |
| --- | --- | --- | --- | --- | --- | --- | --- | --- | --- | --- | --- | --- | --- |
| **1. How should muscle inflammation be treated?** | | | | | | | | | | | | | |
| 1 | | High dose corticosteroids should be used to treat active muscle inflammation at time of treatment induction. | | | - Ruperto(1) - Fisler(2) - Rouster-Stevens(3) - Dawkins(4) - Oddis(5) | 1 B 100% | | | | 21/21 | | |  |
| 1a | | **Adult specific recommendation:**  Oral prednisolone at a dose of 0.5-1mg/kg/day, usually 40-60mg) is recommended. | | | - Oddis(5) - Dawkins(4) | 1 B 100% | | | | 21/21 | | |  |
| 1b | | **Paediatric specific recommendation:** Oral prednisolone at a dose of 1-2mg/kg/day or intravenous methylprednisolone pulses 30mg/kg/day, maximum 1g daily IV dose is recommended. | | | - Ruperto(1) - Fisler(2) - Rouster-Stevens(3) - Dawkins(4) | 1 B 100% | | | | 11/11 | | |  |
| 1c | | Intravenous methylprednisolone is to be considered, especially when there are concerns about gastrointestinal absorption. Use of intravenous methylprednisolone may allow increased therapeutic effect and less toxicity compared to oral corticosteroids. | | | - Fisler(2) - Rouster-Stevens(3) - Matsubara(6) - Al-Mayouf(7) | 2 B 96% | | | | 24/25 | | |  |
| 2 | | Oral prednisolone should be tapered according to clinical response. | | | - Ruperto(1) - Giancane(8) - Oddis(5) - Dawkins(4) | 1 B 100% | | | | 21/21 | | |  |
| 3 | | Disease modifying anti-rheumatic drugs should be used to reduce muscle inflammation, achieve clinical remission, and reduce steroid burden. | | | - Ueno(9) - Keyßer(10) - Ruperto(1) - Casal-Dominguez(11) - Bohan(12) - Villalba(13) - Newman(14) | 1 C 100% | | | | 20/20 | | |  |
| 3a | | **Paediatric specific recommendation:**  Early, complete control of muscle weakness and inflammation should be sought in juvenile-onset IIM, with the aim of improving outcomes and reducing disease-related complications. | | | - Kim(15) - Fisler(2) - Ramanan(16) | 1 B 100% | | | | 20/20 | | |  |
| 3b | | **Paediatric specific recommendation:** A combination of high dose corticosteroid and methotrexate should be used as first-line treatment in most cases. | | | - Ruperto(1) - Al-Mayouf(7) - Miller(17) - Fischer(18) - Ramanan(16) - Fisler(2) | 1 B 100% | | | | 19/19 | | |  |
| 3c | | **Paediatric specific recommendation: A** combination of prednisolone and methotrexate, as opposed to prednisolone and ciclosporin, should be used for the treatment of juvenile-onset IIM as this has a more favourable side effect profile. | | | - Ruperto(1) | 1 B 100% | | | | 18/18 | | |  |
| 3d | | **Paediatric specific recommendation:**  Mycophenolate mofetil is to be considered as a treatment option to improve skin and muscle disease. | | | - Rouster-Stevens(19) - Dagher(20) | 2 C 100% | | | | 18/18 | | |  |
| 3e | | **Adult specific recommendation:**  Methotrexate, azathioprine, tacrolimus, ciclosporin and mycophenolate mofetil are to be considered for the treatment of active myositis and long-term maintenance of disease remission. | | | - Vencovsky(21) - Villalba(13) - Ibrahim(22) - Bunch(23) - Bunch(24) - Joffe(25) - Yu(26) - Yokoyama(27) - Rowin(28) - Danieli(29) - Oddis(30) | 2 C 96% | | | | 23/24 | | |  |
| 4 | | Intravenous immunoglobulin should be considered as a treatment of severe and/or refractory muscle inflammation. | | | - Dalakas(31) - Cherin(32) - Lam(33) - Kampylafka(34) | 1 B 100% | | | | 21/21 | | |  |
| 5 | | Management of IIM should include a safe and appropriate exercise programme led and monitored by a specialist physiotherapist and/or a specialist occupational therapist to improve QoL and function. | | | - Alemo-Munters(35) - Heikilla(36) - Omori(37) - Maillard(38) - Wiesinger(39) | 1 B 96% | | | | 25/26 | | |  |
| 6 | | Rituximab is to be considered as a treatment option for refractory myositis and may be particularly effective in:  a. Juvenile-onset disease  b. Patients with a positive myositis autoantibody profile  c. Patients with lower burden of disease damage. | | | - Oddis(40) - Aggarwal(41) - de Souza(42) - Reed(43) | 2 A 100% | | | | 25/25 | | |  |
| 7 | | Cyclophosphamide should be considered as a treatment option for severe and/or refractory IIM. | | | - Deakin(44) - Cronin(45) - Nagappa(46) - Bae(47) | 1 B 100% | | | | 21/21 | | |  |
| 8 | | **Adult specific recommendation:**  Abatacept is to be considered as a treatment option in refractory adult IIM. | | | - Tjärnlund(48) | 2 B 100% | | | | 24/24 | | |  |
| **2. How should IIM-related skin manifestations be treated?** | | | | | | | | | | | | | |
| 1 | | Rituximab is to be considered for the treatment of refractory skin disease. | | | - Aggarwal(49) | 2 B 100% | | | | 25/25 | | |  |
| 2 | | IVIg should be considered for the treatment of refractory skin disease. | | | - Kampylafka(34) - Lam 2011(33) - Dalakas(31) | 1 B 100% | | | | 25/25 | | |  |
| 3 | | Sun avoidance and use of high factor sunblock is to be considered to reduce likelihood of a disease flare affecting skin or muscle. | | | - Mamyrova(50) | 2 C 100% | | | | 26/26 | | |  |
| 3 | | **Paediatric specific recommendation:**  Systemic immunosuppressive drugs are to be considered for the treatment of ongoing skin disease activity, including reduced nailfold capillary density. | | | - Barth(51) - Ravelli(52) - Mathiesen(53) - Bowyer(54) - Fisler(2) - Chriten-Zaech(55) - Pachman(56) - Stringer(57) - Seshradi(58) | 2 C 100% | | | | 18/18 | | |  |
| 5 | | **Paediatric specific recommendation:**  An early increase in treatment is to be considered in patients with persistent skin disease to aid remission and reduce development of calcinosis. | | | - Fisler(2) - Pachman(56) - Stringer(57) - Seshradi(58) | 2 C 100% | | | | 18/18 | | |  |
| **3. How should IIM-related ILD be managed?** | | | | | | | | | | | | | |
| 1 | | **Paediatric specific recommendation:**  Routine assessment of pulmonary function, including measurement of transfer factor (DLCO) in juvenile-onset IIM should be performed, as pulmonary function abnormalities are frequent and may be asymptomatic. | | | - Prestridge(59) - Sanner(60) | 1 B 100% | | | | 19/19 | | |  |
| 2 | | **Adult specific recommendation:**  Interstitial lung disease should be screened for in high risk patients. | | | - Ma(61) - Lilleker(62) | 1 B 100% | | | | 24/24 | | |  |
| 3 | | **Adult specific recommendation:**  In the treatment of rapidly progressive interstitial lung disease (RP-ILD):   1. Induction therapy with high dose steroids is to be considered. 2. The use of ciclosporin or tacrolimus, alongside steroids, is to be considered in patients with RP-ILD. 3. Cyclophosphamide or rituximab therapy is to be considered early, potentially as part of the induction regimen. | | | Evidence for part 1 (steroids):   - Tillie-Leblond(63) - Kotani(64) - Kameda(65)   Evidence for part 2 (CYA/TAC):   - Miyake(66) - Takada(67) - Ideura(68) - Kotani(69) - Isoda(70) - Go(71) - Kotani(64)   Evidence for part 3 (cyclo/RTX):  **Cyclo**   - Marie(72) - Schnabel(73)   **RTX**   - Sem(74) - Sharp(75) | 2 C 96% | | | | 23/24 | | |  |
| 4 | | **Adult specific recommendation:**  In the treatment of chronic IIM-associated interstitial lung disease:   1. Immunosuppression using steroids with or without a single DMARD (azathioprine, ciclosporin, tacrolimus, mycophenolate) is to be considered. 2. Rituximab or cyclophosphamide is to be considered in treatment-resistant patients. | | | Evidence for part 1 (Steroids and DMARD):  **Steroids**   - Ideura(68) - Marie(76) - Marie(77) - Marie(72) - Marie(78) - Marie(79) - Koreeda(80)   **MMF**   - Saketkoo(81) - Morganroth(82)   **AZA**   - Marie(76) - Marie(77) - Marie(78) - Marie(79)   **CYA/TAC**   - Takada(67) - Ideura(68) - Marie(76) - Ingegnoli(83) - Koreeda(80) - Wilkes(84) - Kotani(69) - Oddis(30) - Witt(85) - Yokoyama(27) - Cavagna(86) - Labirua-Iturburu(87)   Evidence for part 2 (cyclo/RTX):  **Cyclo**   - Yamasaki(88) - Marie(76) - Marie(77) - Marie(72) - Marie(78) - Ingegnoli(83) - Marie(79) - Yamasaki(88) - Bombardieri(89) - Kameda(65) - Mira-Avendano(90)   **RTX**   - Sem(74) - Keir(91) - Marie(92) | 2 C 100% | | | | 24/24 | | |  |
| **4. What management steps should be taken to reduce fracture risk in IIM patients?** | | | | | | | | | | | | | |
| 1 | | **Adult specific recommendation:**  A bone health assessment should be performed, regardless of corticosteroid therapy, and appropriate management instigated. | | | - Gupta(93) - Ng(94) | 1. B 100% | | | | 24/24 | | |  |
| **5. What are key IIM prognostic and management factors that should be considered for children with IIM?** | | | | | | | | | | | | | |
| 1 | | **Paediatric specific recommendation:**  Juvenile-onset IIM should be managed by paediatric specialists; it differs from adult onset DM in several ways including greater presence of subcutaneous calcification, less disease damage, lack of association with cancer, increased risk of vasculitis, and different autoantibody associations. | | | - Na(95) - Rider(96) - Dawkins(97) - Patwardhan(98) - Rider(99) - Shah(100) - Torres(101) | 1 C 95% | | | | 20/21 | | |  |
| 2 | | **Paediatric specific recommendation:**  Shorter time to diagnosis is associated with improved disease outcome. Therefore, early referral to a specialist service is to be considered. | | | - Mathiesen(53) - Huber(102) - Seshradi(58) - Mathiesen(103) - Marie(104) | 2 C 100% | | | | 20/20 | | |  |
| 3 | | **Paediatric specific recommendation:**  Age-specific considerations should be taken into account when using tools that measure muscle strength, function and quality of life. | | | - Sanner(105) - [Quiñones](https://onlinelibrary.wiley.com/action/doSearch?ContribAuthorStored=Qui%C3%B1ones%2C+Rebecca)(106) - Takken(107) - Rennebohn(108) | 1 B 100% | | | | 20/20 | | |  |
| 4 | | **Paediatric specific recommendation:**  Healthcare professionals should look for signs of CTD overlap, which is associated with increased risk of mortality. | | | - Dawkins(97) - Shah(100) - Huber(102) | 1 C 89% | | | | 16/18 | | |  |
| 5 | | **Paediatric specific recommendation:**  Patients with juvenile-onset IIM should be assessed for calcinosis. | | | - Okong'o(109) - Tansley(110) - Gunawardena(111) - Mathiesen(53) - Orandi(112) - Saini(113) - Sallum(114) - Martin(115) | 1 C 100% | | | | 20/20 | | |  |
| **6. Is autoantibody testing useful in people with IIM?** | | | | | | | | | | | | | |
| 1 | Patients should be tested for myositis auto-antibodies. | | | - Rider(99) - Shah(100) - Tansley(110) - Tansley(116) - Betteridge(117) - Huber(102) - Habers(118) | | 1 B 100% | | | 25/25 | | |  |  |
| **7. How should cancer be screened for in people with an IIM?** | | | | | | | | | | | | | |
| 1 | | **Paediatric specific recommendation:**  Routine screening for cancer is not warranted in juvenile-onset IIM. | | | - Gunawardena(119) - Sato(120) - Na(95) - Dawkins(97) | 1 B 100% | | | | 19/19 | | |  |
| 2 | | **Adult specific recommendation:**  The risk of cancer should be considered in all patients, and screening should be particularly considered in those with the following risk factors:   1. Older age at onset 2. Male gender 3. Dysphagia 4. Cutaneous necrosis 5. Resistance to immunosuppressive therapy 6. Rapid disease onset 7. Positive anti-TIF1-gamma autoantibodies 8. Positive anti-NXP2 autoantibodies 9. Negative for known myositis-specific autoantibodies. | | | - Gunawardena(119) - Sato(120) - So(121) - Antiochos(122) - Stockton(123) - Andras(124) - Hill(125) - Wang(126) - Neri(127) - Chen(128) - Marie(129) - Lu(130) - Ponyi(131) - Azuma(132) - Basset-Seguin(133) - Sparsa(134) - Burnouf(135) - Leow(136) - Fardet(137) - Targoff(138) - Kaji(139) - Chinoy(140) - Fujikawa(141) - Trallero-Araguas(142) - Bodoki(143) - Ichimura(144) - Fiorentino(145) - Yang(146) - Oldroyd(147) | 1 B 100% | | | | 23/23 | | |  |
| **8. How should IIM treatment during pregnancy and the breastfeeding period be amended?** | | | | | | | | | | | | | |
| 1 | | Those wishing to conceive should be advised to plan conception whilst their disease is well controlled. | | | - Zhong(148) - Vancsa(149) - Gutierrez(150) | 1 B 100% | | | | 25/25 | | |  |
| 2 | | Pregnancy should be managed in conjunction with maternal medicine specialists. | | | - Zhong(148) - Kolstad(151) | 1 B 96% | | | | 23/24 | | |  |
| 5 | | Increased vigilance is required post-partum - patients may be at risk of disease flare. | | | - Pinal-Fernandez(152) | 1 C 96% | | | | 23/24 | | |  |
| **9. How should IIM-related cardiovascular disease be assessed for and treated?** | | | | | | | | | | | | | |
| 1 | | **Adult specific recommendation:**  Patients should undergo a regular cardiovascular risk assessment | | | - Limaye(153) - Linos(154) | 1 C 100% | | | | 24/24 | | |  |
| 2 | | **Paeds specific recommendation:**  Assessment and management of cardiovascular risk factors is to be considered, including hypertension, obesity or metabolic abnormalities (lipids / insulin resistance). | | | - Coyle(155) - Eimer(156) - Silverberg(157) | 2 C 100% | | | | 19/19 | | |  |
| **10. How should cardiac involvement in IIM be screened for? (Adults)** | | | | | | | | | | | | | |
| 1 | | **Adult specific recommendation:**  Patients should undergo screening for cardiac involvement; serum cardiac damage markers, ECG, echocardiography and cardiac MRI are to be considered. | | | - DeVere(158) - Denbow(159) - Zhang(160) - Danko(161) - Haupt(162) - Kiely(163) - Allanore(164) - Aggarwal(165) - Fisher(166) - Rosenbohm(167) - Diederichsen(168) - Lilleker(169) | 2 B 100% | | | | 25/25 | | |  |
| 2 | | **Adult specific recommendation:**  Cardiac troponin I (not Cardiac troponin T) should be used as the preferred serum marker for screening and monitoring cardiac involvement. | | | - Kiely(163) - Aggarwal(165) - Lilleker(169) - Erlacher(170) | 1 B 100% | | | | 24/24 | | |  |
| 3 | | **Paeds specific recommendation:**  Screening for cardiac involvement in patients with juvenile-onset IIM with ECG and echocardiogram is to be considered | | | - Rider(99) - Eimer(156) - Cantez(171) - Schwartz(172) - Barth(173) | 2 C 100% | | | | 18/18 | | |  |
| **11. How should IIM-related dysphagia be screened for and managed?** | | | | | | | | | | | | | |
| 1 | Routine assessment of dysphagia is to be considered in all patients | | | | - Azola(174) - Chiu(175) - Oh(176) - Casal-Dominguez(177) - Williams(178) - Mugii(179) - Mulcahy(180) - Torres(101) - Taieb(181) - Danko(161) - Marie(182) - Sultan(183) | | | 2 C 92% | | 23/25 | | |  |
| 2 | Swallowing assessment and involvement of speech and language therapist/gastroenterology teams is to be considered in those with dysphagia. | | | | - Azola(174) - Oh(176) - Casal-Dominguez(177) - Williams(178) - Mugii(179) - Langdon(184) - Ertekin(185) - Kim(186) - McCann(187) - Ogawa-Momohara(188) | | | 2 C 100% | | 24/24 | | |  |
| 3 | IVIg therapy for active disease and dysphagia resistant to other treatments is to be considered. | | | | - Marie(189) | | | 2 C 100% | | 25/25 | | |  |
| **12. How should quality of life and mental wellbeing be assessed and treated in people with IIM?** | | | | | | | | | | | | | |
| 1 | | Psychological wellbeing and psychiatric comorbidities should be assessed. | | | - De Souza(190) - Wang(191) - Wang(192) | 1 C 92% | | | | 24/26 | | |  |
| 2 | | Psychological wellbeing and health-related quality of life should be routinely assessed using an age appropriate tool. | | | - Armadans-Tremolosa(193) - Apaz(194) - Ponyi(195) - Sultan(183) - Ravelli(52) - Tollisen(196) - Poulsen(197) | 1 B 100% | | | | 26/26 | | |  |
| 3 | | Factors negatively impacting upon health-related quality of life (e.g. skin involvement, pruritis, steroid adverse effects) should be addressed. | | | - Marie(198) - Ponyi(195) - Goreshi(199) - Hundly(200) - Shirani(201) - Takken(107) - Feldon(202) | 1 C 96% | | | | 25/26 | | |  |
| 4 | | **Paediatric specific recommendation:**  Factors negatively impacting upon health-related quality of life in children include pain, muscle weakness, and poor sleep, and should be managed appropriately. | | | - Butbul Aviel(203) - Challa(204) | 1 C 95% | | | | 20/21 | | |  |
| 5 | | Exercise and/or rehabilitation should be encouraged regardless of disease activity, with the aim of improving psychological wellbeing. | | | - Alexanderson(205) | 1 B 96% | | | | 25/26 | | |  |
| 6 | | Where relevant, targeted exercises given by a specialist physiotherapist and/or a specialist occupational therapist to improve grip strength are to be considered, due to the negative impact of poor grip strength on activities of daily living and quality of life. | | | - Regardt(206) | 2 C 96% | | | | 25/26 | | |  |
| **13. What IIM management considerations should be made for certain ethnic groups?** | | | | | | | | | | | | | |
| 1 | | Ethnicity is to be considered when assessing patients; clinical manifestations, associated autoantibodies, and underlying risk factors may vary according to ethnicity. | - Ng(94) - Koh(207) - Pinal-Fernandez(208) - Narang(209) - Satoh(210) - O’Hanlon(211) - O’Hanlon(212) - Reed(213) | | | | 2 C 96% | | | | 23/24 | | |

**References**

1. Ruperto N, Pistorio A, Oliveira S, Zulian F, Cuttica R, Ravelli A, et al. Prednisone versus prednisone plus ciclosporin versus prednisone plus methotrexate in new-onset juvenile dermatomyositis: a randomised trial. Lancet (London, England) [Internet]. 2016 Feb 13;387(10019):671–8. Available from: http://www.ncbi.nlm.nih.gov/pubmed/26645190

2. Fisler RE, Liang MG, Fuhlbrigge RC, Yalcindag A, Sundel RP. Aggressive management of juvenile dermatomyositis results in improved outcome and decreased incidence of calcinosis. J Am Acad Dermatol. 2002 Oct 1;47(4):505–11.

3. Rouster-Stevens KA, Gursahaney A, Ngai K, Daru JA, Pachman LM. Pharmacokinetic study of oral prednisolone compared with intravenous methylprednisolone in patients with juvenile dermatomyositis. Arthritis Rheum [Internet]. 2008 Feb 15 [cited 2020 Jan 14];59(2):222–6. Available from: http://doi.wiley.com/10.1002/art.23341

4. Dawkins MA, Jorizzo JL, Walker FO, Albertson D, Sinal SH, Hinds A. Dermatomyositis: A dermatology-based case series. J Am Acad Dermatol. 1998;38(3):397–404.

5. Oddis C V, Medsger TA. Relationship between serum creatine kinase level and corticosteroid therapy in polymyositis-dermatomyositis. J Rheumatol [Internet]. 1988 [cited 2020 Jan 14];15(5):807–11. Available from: http://www.ncbi.nlm.nih.gov/pubmed/3172094

6. Matsubara S, Sawa Y, Takamori M, Yokoyama H, Kida H. Pulsed intravenous methylprednisolone combined with oral steroids as the initial treatment of inflammatory myopathies. Vol. 57, Journal of Neurology, Neurosurgery and Psychiatry. BMJ Publishing Group; 1994. p. 1008.

7. Al-Mayouf S, Al-Mazyed A A, Bahabri S. Efficacy of early treatment of severe juvenile dermatomyositis with intravenous methylprednisolone and methotrexate. Clin Rheumatol. 2000;19(2):138–41.

8. Giancane G, Lavarello C, Pistorio A, Oliveira SK, Zulian F, Cuttica R, et al. The PRINTO evidence-based proposal for glucocorticoids tapering/discontinuation in new onset juvenile dermatomyositis patients. Pediatr Rheumatol. 2019;17(1):1–11.

9. Ueno KI, Shimojima Y, Kishida D, Sekijima Y, Ikeda SI. Advantage of administering tacrolimus for improving prognosis of patients with polymyositis and dermatomyositis. Int J Rheum Dis. 2016;19(12):1322–30.

10. Keyßer G, Zierz S, Kornhuber M. Treatment of adult idiopathic inflammatory myopathies with conventional immunosuppressive drugs: Results of a retrospective study. Z Rheumatol. 2019;78(2):183–9.

11. Casal-Dominguez M, Pinal-Fernandez I, Huapaya J, Albayda J, Paik JJ, Johnson C, et al. Efficacy and adverse effects of methotrexate compared with azathioprine in the antisynthetase syndrome. Clin Exp Rheumatol. 2019;37(5):858–61.

12. Bohan A, Peter JB, Bowman RL, Pearson CM. Computer-assisted analysis of 153 patients with polymyositis and dermatomyositis. Medicine (Baltimore) [Internet]. 1977 Jul [cited 2017 Mar 24];56(4):255–86. Available from: http://www.ncbi.nlm.nih.gov/pubmed/327194

13. L V, JE H, EM A, JB S, MF G, RL L, et al. Treatment of Refractory Myositis: A Randomized Crossover Study of Two New Cytotoxic Regimens. Arthritis Rheum [Internet]. 1998 [cited 2020 Jun 29];41(3). Available from: https://pubmed.ncbi.nlm.nih.gov/9506565/

14. Newman ED, Scott DW. The use of low-dose oral methotrexate in the treatment of polymyositis and dermatomyositis. J Clin Rheumatol. 1995;1(2):99–102.

15. Kim S, El-Hallak M, Dedeoglu F, Zurakowski D, Fuhlbrigge RC, Sundel RP. Complete and sustained remission of juvenile dermatomyositis resulting from aggressive treatment. Arthritis Rheum. 2009;60(6):1825–30.

16. Ramanan A V., Campbell-Webster N, Ota S, Parker S, Tran D, Tyrrell PN, et al. The effectiveness of treating juvenile dermatomyositis with methotrexate and aggressively tapered corticosteroids. Arthritis Rheum. 2005;52(11):3570–8.

17. Miller LC, Sisson BA, Tucker LB, Denardo BA, Schaller JG. Methotrexate treatment of recalcitrant childhood dermatomyositis. Arthritis Rheum. 1992;35(10):1143–9.

18. Fischer TJ, Rachelefsky GS, Klein RB, Paulus HE, Stiehm ER. Childhood Dermatomyositis and Polymyositis: Treatment With Methotrexate and Prednisone. Am J Dis Child [Internet]. 1979 Apr 1 [cited 2021 Jul 1];133(4):386–9. Available from: https://jamanetwork.com/journals/jamapediatrics/fullarticle/508444

19. Rouster-Stevens KA, Morgan GA, Wang D, Pachman LM. Mycophenolate mofetil: a possible therapeutic agent for children with juvenile dermatomyositis. Arthritis Care Res (Hoboken) [Internet]. 2010 Oct [cited 2014 Feb 6];62(10):1446–51. Available from: http://www.pubmedcentral.nih.gov/articlerender.fcgi?artid=2952049&tool=pmcentrez&rendertype=abstract

20. Dagher R, Desjonquères M, Duquesne A, Quartier P, Bader-Meunier B, Fischbach M, et al. Mycophenolate mofetil in juvenile dermatomyositis: a case series. Rheumatol Int [Internet]. 2012 Mar [cited 2014 Feb 6];32(3):711–6. Available from: http://www.ncbi.nlm.nih.gov/pubmed/21152922

21. Vencovský J, Jarošová K, Macháček S, Studýnková J, Kafkov́ J, Bartůňkov́ J, et al. Cyclosporine A versus methotrexate in the treatment of polymyositis and dermatomyositis. Scand J Rheumatol [Internet]. 2000 [cited 2020 Jun 29];29(2):95–102. Available from: https://pubmed.ncbi.nlm.nih.gov/10777122/

22. Ibrahim F, Choy E, Gordon P, Doré CJ, Hakim A, Kitas G, et al. Concise report Second-line agents in myositis: 1-year factorial trial of additional immunosuppression in patients who have partially responded to steroids. Rheumatology [Internet]. 2015 [cited 2020 Jun 29];54:1050–5.

23. Bunch TW, Worthington JW, Combs JJ, Ilstrup DM, Engel AG. Azathioprine with prednisone for polymyositis. A controlled, clinical trial. Ann Intern Med [Internet]. 1980 [cited 2020 Jun 29];92(3):365–9. Available from: https://pubmed.ncbi.nlm.nih.gov/6986827/

24. Bunch TW. Prednisone and azathioprine for polymyositis. Long‐term followup. Arthritis Rheum [Internet]. 1981 [cited 2020 Jun 29];24(1):45–8. Available from: https://pubmed.ncbi.nlm.nih.gov/7008799/

25. Joffe MM, Love LA, Leff RL, Fraser DD, Targoff IN, Hicks JE, et al. Drug therapy of the idiopathic inflammatory myopathies: predictors of response to prednisone, azathioprine, and methotrexate and a comparison of their efficacy. Am J Med [Internet]. 1993 [cited 2020 Jun 29];94(4):379–87. Available from: https://pubmed.ncbi.nlm.nih.gov/8386437/

26. Yu KH, Wu YJJ, Kuo CF, See LC, Shen YM, Chang HC, et al. Survival analysis of patients with dermatomyositis and polymyositis: Analysis of 192 Chinese cases. Clin Rheumatol [Internet]. 2011 Dec [cited 2020 Jun 29];30(12):1595–601. Available from: https://pubmed.ncbi.nlm.nih.gov/21915609/

27. Yokoyama Y, Furuta S, Ikeda K, Hirose K, Nakajima H. Corticosteroid-sparing effect of tacrolimus in the initial treatment of dermatomyositis and polymyositis. Mod Rheumatol [Internet]. 2015 Sep 8 [cited 2020 Jun 29];25(6):888–92. Available from: https://www.tandfonline.com/doi/abs/10.3109/14397595.2015.1029239

28. Rowin J, Amato AA, Deisher N, Cursio J, Meriggioli MN. Mycophenolate mofetil in dermatomyositis: Is it safe? Neurology [Internet]. 2006 Apr 25 [cited 2020 Jun 29];66(8):1245–7. Available from: https://n.neurology.org/content/66/8/1245

29. Danieli MG, Calcabrini L, Calabrese V, Marchetti A, Logullo F, Gabrielli A. Intravenous immunoglobulin as add on treatment with mycophenolate mofetil in severe myositis [Internet]. Vol. 9, Autoimmunity Reviews. Autoimmun Rev; 2009 [cited 2020 Jun 29]. p. 124–7. Available from: https://pubmed.ncbi.nlm.nih.gov/19386287/

30. Oddis C V., Sciurba FC, Elmagd KA, Starzl TE. Tacrolimus in refractory polymyositis with interstitial lung disease. Lancet [Internet]. 1999 May 22 [cited 2020 Jun 30];353(9166):1762–3. Available from: https://www.ncbi.nlm.nih.gov/pmc/articles/PMC2977932/

31. Dalakas MC, Illa I, Dambrosia JM, Soueidan SA, Stein DP, Otero C, et al. A controlled trial of high-dose intravenous immune globulin infusions as treatment for dermatomyositis. N Engl J Med [Internet]. 1993 Dec 30 [cited 2017 Oct 20];329(27):1993–2000. Available from: http://www.ncbi.nlm.nih.gov/pubmed/8247075

32. Cherin P, Pelletier S, Teixeira A, Laforet P, Genereau T, Simon A, et al. Results and long-term followup of intravenous immunoglobulin infusions in chronic, refractory polymyositis: An open study with thirty-five adult patients. Arthritis Rheum [Internet]. 2002 [cited 2020 Jun 30];46(2):467–74. Available from: https://pubmed.ncbi.nlm.nih.gov/11840450/

33. Lam CG, Manlhiot C, Pullenayegum EM, Feldman BM. Efficacy of intravenous Ig therapy in juvenile dermatomyositis. Ann Rheum Dis. 2011 Dec;70(12):2089–94.

34. Kampylafka EI, Kosmidis ML, Panagiotakos DB, Dalakas M, Moutsopoulos HM, Tzioufas AG. The effect of intravenous immunoglobulin (IVIG) treatment on patients with dermatomyositis: a 4-year follow-up study. Clin Exp Rheumatol [Internet]. [cited 2020 Jan 15];30(3):397–401. Available from: http://www.ncbi.nlm.nih.gov/pubmed/22510247

35. Alemo Munters L, Dastmalchi M, Andgren V, Emilson C, Bergegård J, Regardt M, et al. Improvement in Health and Possible Reduction in Disease Activity Using Endurance Exercise in Patients With Established Polymyositis and Dermatomyositis: A Multicenter Randomized Controlled Trial With a 1-Year Open Extension Followup. Arthritis Care Res (Hoboken) [Internet]. 2013 Dec [cited 2017 Oct 20];65(12):1959–68. Available from: http://www.ncbi.nlm.nih.gov/pubmed/23861241

36. Heikkillä S, Viitanen J V., Kautiainen H, Rajamäki T, Mäntyvuo P, Harju T. Rehabilitation in Myositis. Physiotherapy. 2001;87(6):301–9.

37. Omori CH, Silva CAA, Sallum AME, Rodrigues Pereira RM, Luciade Sa Pinto A, Roschel H, et al. Exercise training in juvenile dermatomyositis. Arthritis Care Res (Hoboken). 2012 Aug;64(8):1186–94.

38. Maillard SM, Jones R, Owens CM, Pilkington C, Woo PM, Wedderburn LR, et al. Quantitative assessments of the effects of a single exercise session on muscles in juvenile dermatomyositis. Arthritis Rheum. 2005 Aug;53(4):558–64.

39. Wiesinger GF, Quittan M, Aringer M, Seeber A, Volc-Platzer B, Smolen J, et al. Improvement of physical fitness and muscle strength in polymyositis/dermatomyositis patients by a training programme. Br J Rheumatol. 1998 Feb;37(2):196–200.

40. Oddis C V, Reed AM, Aggarwal R, Rider LG, Ascherman DP, Levesque MC, et al. Rituximab in the treatment of refractory adult and juvenile dermatomyositis and adult polymyositis: a randomized, placebo-phase trial. Arthritis Rheum [Internet]. 2013 Feb [cited 2016 Jun 23];65(2):314–24. Available from: http://www.ncbi.nlm.nih.gov/pubmed/23124935

41. Aggarwal R, Bandos A, Reed AM, Ascherman DP, Barohn RJ, Feldman BM, et al. Predictors of clinical improvement in rituximab-treated refractory adult and juvenile dermatomyositis and adult polymyositis. Arthritis Rheumatol (Hoboken, NJ) [Internet]. 2014 Mar [cited 2014 Mar 20];66(3):740–9. Available from: http://www.ncbi.nlm.nih.gov/pubmed/24574235

42. de Souza FHC, Miossi R, de Moraes JCB, Bonfá E, Shinjo SK. Favorable rituximab response in patients with refractory idiopathic inflammatory myopathies. Adv Rheumatol (London, England). 2018;58(1):31.

43. Reed AM, Crowson CS, Hein M, de Padilla CL, Olazagasti JM, Aggarwal R, et al. Biologic predictors of clinical improvement in rituximab-treated refractory myositis. BMC Musculoskelet Disord [Internet]. 2015 Dec 17 [cited 2020 Jan 14];16(1):257. Available from: http://bmcmusculoskeletdisord.biomedcentral.com/articles/10.1186/s12891-015-0710-3

44. Deakin C, Campanilho-Marques R, Simou S, Moraitis E, Wedderburn L, Pullenayegum E, et al. Efficacy and Safety of Cyclophosphamide Treatment in Severe Juvenile Dermatomyositis Shown by Marginal Structural Modeling. Arthritis Rheumatol. 2018;70(5):785–93.

45. Cronin ME, Miller FW, Hicks JE, Dalakas M, Plotz PH. The failure of intravenous cyclophosphamide therapy in refractory idiopathic inflammatory myopathy. J Rheumatol [Internet]. 1989 Sep [cited 2020 Jan 14];16(9):1225–8. Available from: http://www.ncbi.nlm.nih.gov/pubmed/2681763

46. Nagappa M, Taly AB, Sinha S, Gayathri N, Bindu PS, Mahadevan A, et al. Efficacy and limitations of pulse cyclophosphamide therapy in polymyositis and dermatomyositis. J Clin Neuromuscul Dis. 2013 Jun;14(4):161–8.

47. Bae S, Charles-Schoeman C. Oral cyclophosphamide in treatment of patients with refractory idiopathic inflammatory myopathies: a retrospective observational study. Clin Rheumatol. 2018 Aug 1;37(8):2113–23.

48. Tjärnlund A, Tang Q, Wick C, Dastmalchi M, Mann H, Studýnková JT, et al. Abatacept in the treatment of adult dermatomyositis and polymyositis: A randomised, phase IIb treatment delayed-start trial. Ann Rheum Dis. 2018;77(1):55–62.

49. Aggarwal R, Loganathan P, Koontz D, Qi Z, Reed AM, Oddis C V. Cutaneous improvement in refractory adult and juvenile dermatomyositis after treatment with rituximab. Rheumatol (United Kingdom). 2017;56(2):247–54.

50. Mamyrova G, Rider LG, Ehrlich A, Jones O, Pachman LM, Nickeson R, et al. Environmental factors associated with disease flare in juvenile and adult dermatomyositis. Rheumatol (United Kingdom). 2017;56(8):1342–7.

51. Barth Z, Witczak BN, Flatø B, Koller A, Sjaastad I, Sanner H. Assessment of Microvascular Abnormalities by Nailfold Capillaroscopy in Juvenile Dermatomyositis After Medium- to Long-Term Followup. Arthritis Care Res [Internet]. 2018 May 1 [cited 2021 Jul 2];70(5):768–76. Available from: https://pubmed.ncbi.nlm.nih.gov/28805016/

52. Ravelli A, Trail L, Ferrari C, Ruperto N, Pistorio A, Pilkington C, et al. Long-term outcome and prognostic factors of juvenile dermatomyositis: A multinational, multicenter study of 490 patients. Arthritis Care Res. 2010;62(1):63–72.

53. Mathiesen PR, Zak M, Herlin T, Nielsen SM. Clinical features and outcome in a Danish cohort of juvenile dermatomyositis patients. Clin Exp Rheumatol. 2010;28(5):782–9.

54. Bowyer SL, Blane CE, Sullivan DB, Cassidy JT. Childhood dermatomyositis: Factors predicting functional outcome and development of dystrophic calcification. J Pediatr. 1983;103(6):882–8.

55. Christen-Zaech S, Seshadri R, Sundberg J, Paller AS, Pachman LM. Persistent association of nailfold capillaroscopy changes and skin involvement over thirty-six months with duration of untreated disease in patients with juvenile dermatomyositis. Arthritis Rheum [Internet]. 2008 Feb [cited 2021 Jul 2];58(2):571–6. Available from: /pmc/articles/PMC2830145/

56. Pachman LM, Veis A, Stock S, Abbott K, Vicari F, Patel P, et al. Composition of calcifications in children with juvenile dermatomyositis: Association with chronic cutaneous inflammation. Arthritis Rheum. 2006 Oct;54(10):3345–50.

57. Stringer E, Singh-Grewal D, Feldman BM. Predicting the course of juvenile dermatomyositis: Significance of early clinical and laboratory features. Arthritis Rheum [Internet]. 2008 Nov [cited 2020 Jan 15];58(11):3585–92. Available from: http://doi.wiley.com/10.1002/art.23960

58. Seshadri R, Feldman BM, Ilowite N, Cawkwell G, Pachman LM. The role of aggressive corticosteroid therapy in patients with juvenile dermatomyositis: A propensity score analysis. Arthritis Care Res. 2008;59(7):989–95.

59. Prestridge A, Morgan G, Ferguson L, Huang CC, Pachman LM. Pulmonary function tests in idiopathic inflammatory myopathy: Association with clinical parameters in children. Arthritis Care Res. 2013;65(9):1424–31.

60. Sanner H, Aaløkken TM, Gran JT, Sjaastad I, Johansen B, Flatø B. Pulmonary outcome in juvenile dermatomyositis: A case-control study. Ann Rheum Dis [Internet]. 2011 Jan [cited 2021 Jul 2];70(1):86–91. Available from: https://pubmed.ncbi.nlm.nih.gov/20805295/

61. Ma X, Chen Z, Hu W, Guo Z, Wang Y, Kuwana M, et al. Clinical and serological features of patients with dermatomyositis complicated by spontaneous pneumomediastinum. Clin Rheumatol. 2016;35(2):489–93.

62. Lilleker JB, Vencovsky J, Wang G, Wedderburn LR, Diederichsen LP, Schmidt J, et al. The EuroMyositis registry: an international collaborative tool to facilitate myositis research. Ann Rheum Dis [Internet]. 2018 Jan [cited 2018 Apr 9];77(1):30–9. Available from: http://www.ncbi.nlm.nih.gov/pubmed/28855174

63. Tillie-Leblond I, Wislez M, Valeyre D, Crestani B, Rabbat A, Israel-Biet D, et al. Interstitial lung disease and anti-Jo-1 antibodies: Difference between acute and gradual onset. Thorax [Internet]. 2008 Jan [cited 2020 Jun 29];63(1):53–9. Available from: https://pubmed.ncbi.nlm.nih.gov/17557770/

64. Kotani T, Makino S, Takeuchi T, Kagitani M, Shoda T, Hata A, et al. Early intervention with corticosteroids and cyclosporin A and 2-hour postdose blood concentration monitoring improves the prognosis of acute/subacute interstitial pneumonia in dermatomyositis. J Rheumatol. 2008;35(2).

65. Kameda H, Nagasawa H, Ogawa H, Sekiguchi N, Takei H, Tokuhira M, et al. Combination therapy with corticosteroids, cyclosporin A, and intravenous pulse cyclophosphamide for acute/subacute interstitial pneumonia in patients with dermatomyositis. J Rheumatol. 2005;32(9).

66. Miyake S, Ohtani Y, Sawada M, Inase N, Miyazaki Y, Takano S, et al. Usefulness of cyclosporine A on rapidly progressive interstitial pneumonia in dermatomyositis. Sarcoidosis, Vasc Diffus lung Dis Off J WASOG [Internet]. 2002 Jun 1 [cited 2020 Jun 29];19(2):128–33. Available from: http://www.ncbi.nlm.nih.gov/pubmed/12102608

67. Takada K, Nagasaka K, Miyasaka N. Polymyositis/dermatomyositis and interstitial lung disease: A new therapeutic approach with T-cell-specific immunosuppressants [Internet]. Vol. 38, Autoimmunity. Autoimmunity; 2005 [cited 2020 Jun 29]. p. 383–92. Available from: https://pubmed.ncbi.nlm.nih.gov/16227154/

68. Ideura G, Hanaoka M, Koizumi T, Fujimoto K, Shimojima Y, Ishii W, et al. Interstitial lung disease associated with amyopathic dermatomyositis: Review of 18 cases. Respir Med [Internet]. 2007 Jul [cited 2020 Jun 29];101(7):1406–11. Available from: https://pubmed.ncbi.nlm.nih.gov/17353121/

69. Kotani T, Takeuchi T, Makino S, Hata K, Yoshida S, Nagai K, et al. Combination with corticosteroids and cyclosporin-A improves pulmonary function test results and chest HRCT findings in dermatomyositis patients with acute/subacute interstitial pneumonia. Clin Rheumatol [Internet]. 2011 Aug [cited 2020 Jun 29];30(8):1021–8. Available from: http://www.ncbi.nlm.nih.gov/pubmed/21340495

70. Isoda K, Takeuchi T, Kotani T, Hata K, Shoda T, Ishida T, et al. Pre-treatment ferritin level and alveolar-arterial oxygen gradient can predict mortality rate due to acute/subacute interstitial pneumonia in dermatomyositis treated by cyclosporine A/glucocorticosteroid combination therapy: A case control study. PLoS One [Internet]. 2014 Feb 21 [cited 2020 Jun 29];9(2). Available from: https://pubmed.ncbi.nlm.nih.gov/24586910/

71. Go DJ, Park JK, Kang EH, Kwon HM, Lee YJ, Song YW, et al. Survival benefit associated with early cyclosporine treatment for dermatomyositis-associated interstitial lung disease. Rheumatol Int. 2016 Jan 1;36(1):125–31.

72. Marie I, Hatron PY, Dominique S, Cherin P, Mouthon L, Menard JF. Short-term and long-term outcomes of interstitial lung disease in polymyositis and dermatomyositis: a series of 107 patients. Arthritis Rheum [Internet]. 2011;63:3439–47. Available from: http://www.ncbi.nlm.nih.gov/pubmed/21702020

73. Schnabel A, Reuter M, Biederer J, Richter C, Gross WL. Interstitial lung disease in polymyositis and dermatomyositis: Clinical course and response to treatment. Semin Arthritis Rheum [Internet]. 2003 [cited 2020 Jun 29];32(5):273–84. Available from: https://pubmed.ncbi.nlm.nih.gov/12701038/

74. Sem M, Molber Ø, Lund MB, Gran JT. Rituximab treatment of the anti-synthetase syndrome - A retrospective case series. Rheumatology. 2009;48(8):968–71.

75. Sharp C, McCabe M, Dodds N, Edey A, Mayers L, Adamali H, et al. Rituximab in autoimmune connective tissue disease-associated interstitial lung disease. Rheumatol (United Kingdom) [Internet]. 2016 Jul 1 [cited 2020 Oct 6];55(7):1318–24. Available from: https://academic.oup.com/rheumatology/article/55/7/1318/1744705

76. Marie I, Hachulla E, Chérin P, Dominique S, Hatron P-Y, Hellot M-F, et al. Interstitial lung disease in polymyositis and dermatomyositis. Arthritis Care Res (Hoboken). 2002 Dec;47(6):614–22.

77. Marie I, Lahaxe L, Benveniste O, Delavigne K, Adoue D, Mouthon L, et al. Long-term outcome of patients with polymyositis/ dermatomyositis and anti-PM-Scl antibody. Br J Dermatol [Internet]. 2010 Feb [cited 2020 Jun 30];162(2):337–44. Available from: https://pubmed.ncbi.nlm.nih.gov/19845665/

78. Marie I, Josse S, Decaux O, Diot E, Landron C, Roblot P, et al. Clinical manifestations and outcome of anti-PL7 positive patients with antisynthetase syndrome. Eur J Intern Med [Internet]. 2013 Jul [cited 2020 Jun 30];24(5):474–9. Available from: https://pubmed.ncbi.nlm.nih.gov/23375620/

79. Marie I, Josse S, Hatron PY, Dominique S, Hachulla E, Janvresse A, et al. Interstitial lung disease in anti-Jo-1 patients with antisynthetase syndrome. Arthritis Care Res [Internet]. 2013 May [cited 2020 Jun 30];65(5):800–8. Available from: https://pubmed.ncbi.nlm.nih.gov/23203765/

80. Koreeda Y, Higashimoto I, Yamamoto M, Takahashi M, Kaji K, Fujimoto M, et al. Clinical and pathological findings of interstitial lung disease patients with anti-aminoacyl-tRNA synthetase autoantibodies. Intern Med [Internet]. 2010 [cited 2020 Jun 30];49(5):361–9. Available from: https://pubmed.ncbi.nlm.nih.gov/20190466/

81. Saketkoo LA, Espinoza LR. Experience of mycophenolate mofetil in 10 patients with autoimmune-related interstitial lung disease demonstrates promising effects. Am J Med Sci [Internet]. 2009 [cited 2020 Jun 30];337(5):329–35. Available from: https://pubmed.ncbi.nlm.nih.gov/19295413/

82. Morganroth PA, Kreider ME, Werth VP. Mycophenolate mofetil for interstitial lung disease in dermatomyositis. Arthritis Care Res [Internet]. 2010 [cited 2020 Jun 30];62(10):1496–501. Available from: /pmc/articles/PMC3085935/?report=abstract

83. Ingegnoli F, Lubatti C, Ingegnoli A, Boracchi P, Zeni S, Meroni PL. Interstitial lung disease outcomes by high-resolution computed tomography (HRCT) in Anti-Jo1 antibody-positive polymyositis patients: A single centre study and review of the literature. Vol. 11, Autoimmunity Reviews. Elsevier; 2012. p. 335–40.

84. Wilkes MR, Sereika SM, Fertig N, Lucas MR, Oddis C V. Treatment of antisynthetase-associated interstitial lung disease with tacrolimus. Arthritis Rheum. 2005;52(8):2439–46.

85. Witt LJ, Demchuk C, Curran JJ, Strek ME. Benefit of adjunctive tacrolimus in connective tissue disease-interstitial lung disease. Pulm Pharmacol Ther [Internet]. 2016 Feb 1 [cited 2020 Jun 30];36:46–52. Available from: https://pubmed.ncbi.nlm.nih.gov/26762710/

86. Cavagna L, Caporali R, Abdì-Alì L, Dore R, Meloni F, Montecucco C. Cyclosporine in anti-Jo1-positive patients with corticosteroid-refractory interstitial lung disease. J Rheumatol [Internet]. 2013 Apr [cited 2020 Jun 29];40(4):484–92. Available from: https://pubmed.ncbi.nlm.nih.gov/23418387/

87. Labirua-Iturburu A, Selva-O’Callaghan A, Martínez-Gómez X, Trallero-Araguás E, Labrador-Horrillo M, Vilardell-Tarrés M. Calcineurin inhibitors in a cohort of patients with antisynthetase-associated interstitial lung disease. Clin Exp Rheumatol [Internet]. 2013 Mar 7 [cited 2020 Jun 30];31(3):436–9. Available from: https://europepmc.org/article/med/23465087

88. Yamasaki Y, Yamada H, Yamasaki M, Ohkubo M, Azuma K, Matsuoka S, et al. Intravenous cyclophosphamide therapy for progressive interstitial pneumonia in patients with polymyositis/ dermatomyositis. Rheumatology. 2007;46:124–30.

89. Bombardieri S, Hughes GRV, Neri R, Del Bravo P, Del Bono L. Cyclophosphamide in severe polymyositis [Internet]. Vol. 333, Lancet. Lancet; 1989 [cited 2020 Jun 30]. p. 1138–9. Available from: https://pubmed.ncbi.nlm.nih.gov/2566079/

90. Mira-Avendano IC, Parambil JG, Yadav R, Arrossi V, Xu M, Chapman JT, et al. A retrospective review of clinical features and treatment outcomes in steroid-resistant interstitial lung disease from polymyositis/dermatomyositis. Respir Med [Internet]. 2013 Jun [cited 2020 Jun 30];107(6):890–6. Available from: https://pubmed.ncbi.nlm.nih.gov/23517887/

91. Keir GJ, Maher TM, Hansell DM, Denton CP, Ong VH, Singh S, et al. Severe interstitial lung disease in connective tissue disease: Rituximab as rescue therapy. Eur Respir J [Internet]. 2012 Sep 1 [cited 2020 Jun 30];40(3):641–8. Available from: https://pubmed.ncbi.nlm.nih.gov/22282550/

92. Marie I, Dominique S, Janvresse A, Levesque H, Menard JF. Rituximab therapy for refractory interstitial lung disease related to antisynthetase syndrome. Respir Med [Internet]. 2012;106(4):581–7. Available from: http://dx.doi.org/10.1016/j.rmed.2012.01.001

93. Gupta L, Lawrence A, Edavalath S, Misra R. Prevalence and predictors of asymptomatic vertebral fractures in inflammatory myositis. Int J Rheum Dis. 2018;21(3):725–31.

94. Ng KP, Ramos F, Sultan SM, Isenberg DA. Concomitant diseases in a cohort of patients with idiopathic myositis during long-term follow-up. Clin Rheumatol [Internet]. 2009 Aug 23 [cited 2017 Oct 23];28(8):947–53. Available from: http://www.ncbi.nlm.nih.gov/pubmed/19387765

95. Na SJ, Kim SM, Sunwoo IN, Choi YC. Clinical characteristics and outcomes of juvenile and adult dermatomyositis. J Korean Med Sci. 2009;24(4):715–21.

96. Rider LG, Lachenbruch PA, Monroe JB, Ravelli A, Cabalar I, Feldman BM, et al. Damage extent and predictors in adult and juvenile dermatomyositis and polymyositis as determined with the myositis damage index. Arthritis Rheum. 2009;60(11):3425–35.

97. Dawkins MA, Jorizzo JL, Walker FO, Albertson D, Sinal SH, Hinds A. Dermatomyositis: a dermatology-based case series. J Am Acad Dermatol. 1998 Mar;38(3):397–404.

98. Patwardhan A, Rennebohm R, Dvorchik I, Spencer CH. Is juvenile dermatomyositis a different disease in children up to three years of age at onset than in children above three years at onset? A retrospective review of 23 years of a single center’s experience. Pediatr Rheumatol. 2012;10:1–12.

99. Rider LG, Shah M, Mamyrova G, Huber AM, Rice MM, Targoff IN, et al. The myositis autoantibody phenotypes of the juvenile idiopathic inflammatory myopathies. Med (United States). 2013;92(4):223–43.

100. Shah M, Mamyrova G, Targoff IN, Huber AM, Malley JD, Rice MM, et al. The clinical phenotypes of the juvenile idiopathic inflammatory myopathies. Med (United States). 2013;92(1):25–41.

101. Torres C, Belmonte R, Carmona L, Gomez-Reino FJ, Galindo M, Ramos B, et al. Survival, mortality and causes of death in inflammatory myopathies. Autoimmunity [Internet]. 2006;39:205–15. Available from: http://www.ncbi.nlm.nih.gov/pubmed/16769654

102. Huber AM, Mamyrova G, Lachenbruch PA, Lee JA, Katz JD, Targoff IN, et al. Early illness features associated with mortality in the juvenile idiopathic inflammatory myopathies. Arthritis Care Res. 2014;66(5):732–40.

103. Mathiesen P, Hegaard H, Herlin T, Zak M, Pedersen FK, Nielsen S. Long-term outcome in patients with juvenile dermatomyositis: A cross-sectional follow-up study. Scand J Rheumatol. 2012;41(1):50–8.

104. Marie I, Hachulla E, Hatron PY, Hellot MF, Levesque H, Devulder B, et al. Polymyositis and dermatomyositis: short term and longterm outcome, and predictive factors of prognosis. J Rheumatol [Internet]. 2001 Oct [cited 2017 Oct 27];28(10):2230–7. Available from: http://www.ncbi.nlm.nih.gov/pubmed/11669162

105. Sanner H, Sjaastad I, Flatø B. Disease activity and prognostic factors in juvenile dermatomyositis: A long-term follow-up study applying the Paediatric Rheumatology International Trials Organization criteria for inactive disease and the myositis disease activity assessment tool. Rheumatol (United Kingdom). 2014;53(9):1578–85.

106. Quiñones R, Morgan GA, Amoruso M, Field R, Huang CC, Pachman LM. Lack of achievement of a full score on the childhood myositis assessment scale by healthy four-year-olds and those recovering from juvenile dermatomyositis. Arthritis Care Res [Internet]. 2013 Oct [cited 2021 Jun 3];65(10):1697–701. Available from: https://pubmed.ncbi.nlm.nih.gov/23666925/

107. Takken T, Elst E, Spermon N, Helders PJM, Prakken ABJ, van der Net J. The physiological and physical determinants of functional ability measures in children with juvenile dermatomyositis. Rheumatology. 2003;42(4):591–5.

108. Rennebohm RM, Jones K, Huber AM, Ballinger SH, Bowyer SL, Feldman BM, et al. Normal scores for nine maneuvers of the childhood myositis assessment scale. Arthritis Care Res [Internet]. 2004 Jun 15 [cited 2021 Jun 3];51(3):365–70. Available from: https://pubmed.ncbi.nlm.nih.gov/15188320/

109. Okong’o LO, Esser M, Wilmshurst J, Scott C. Characteristics and outcome of children with juvenile dermatomyositis in Cape Town: A cross-sectional study. Pediatr Rheumatol [Internet]. 2016;14(1):1–8. Available from: http://dx.doi.org/10.1186/s12969-016-0118-0

110. Tansley SL, Betteridge ZE, Shaddick G, Gunawardena H, Arnold K, Wedderburn LR, et al. Calcinosis in juvenile dermatomyositis is influenced by both anti-NXP2 autoantibody status and age at disease onset. Rheumatol (United Kingdom) [Internet]. 2014 Dec 1 [cited 2021 Jun 3];53(12):2204–8. Available from: https://pubmed.ncbi.nlm.nih.gov/24987158/

111. Gunawardena H, Wedderburn LR, Chinoy H, Betteridge ZE, North J, Ollier WER, et al. Autoantibodies to a 140-kd protein in juvenile dermatomyositis are associated with calcinosis. Arthritis Rheum [Internet]. 2009 Jun [cited 2021 Jun 3];60(6):1807–14. Available from: https://pubmed.ncbi.nlm.nih.gov/19479859/

112. Orandi AB, Dharnidharka VR, Al-Hammadi N, Baszis KW. Clinical phenotypes and biologic treatment use in juvenile dermatomyositis-associated calcinosis. Pediatr Rheumatol [Internet]. 2018 Dec 29 [cited 2021 Jul 2];16(1). Available from: https://pubmed.ncbi.nlm.nih.gov/30594206/

113. Saini I, Kalaivani M, Kabra SK. Calcinosis in juvenile dermatomyositis: frequency, risk factors and outcome. Rheumatol Int [Internet]. 2016 Jul 1 [cited 2021 Jul 2];36(7):961–5. Available from: https://pubmed.ncbi.nlm.nih.gov/27007612/

114. Sallum AME, Pivato FCMM, Doria-Filho U, Aikawa NE, Liphaus BL, Marie SKN, et al. Risk factors associated with calcinosis of juvenile dermatomyositis. J Pediatr (Rio J). 2008;84(1).

115. Martin N, Krol P, Smith S, Beard L, Pilkington CA, Davidson J, et al. Comparison of children with onset of juvenile dermatomyositis symptoms before or after their fifth birthday in a UK and Ireland juvenile dermatomyositis cohort study. Arthritis Care Res (Hoboken) [Internet]. 2012 Nov 27 [cited 2021 Jul 2];64(11):1665–72. Available from: https://onlinelibrary.wiley.com/doi/10.1002/acr.21753

116. Tansley SL, Simou S, Shaddick G, Betteridge ZE, Almeida B, Gunawardena H, et al. Autoantibodies in juvenile-onset myositis: Their diagnostic value and associated clinical phenotype in a large UK cohort. J Autoimmun. 2017 Nov 1;84:55–64.

117. Betteridge Z, Tansley S, Shaddick G, Chinoy H, Cooper RG, New RP, et al. Frequency, mutual exclusivity and clinical associations of myositis autoantibodies in a combined European cohort of idiopathic inflammatory myopathy patients. J Autoimmun [Internet]. 2019 Jul 1 [cited 2020 Aug 13];101:48–55. Available from: https://pubmed.ncbi.nlm.nih.gov/30992170/

118. Habers GEA, Huber AM, Mamyrova G, Targoff IN, O’Hanlon TP, Adams S, et al. Association of Myositis Autoantibodies, Clinical Features, and Environmental Exposures at Illness Onset with Disease Course in Juvenile Myositis. Arthritis Rheumatol [Internet]. 2016 Mar 1 [cited 2021 Sep 17];68(3):761–8. Available from: https://pubmed.ncbi.nlm.nih.gov/26474155/

119. Gunawardena H, Wedderburn LR, North J, Betteridge Z, Dunphy J, Chinoy H, et al. Clinical associations of autoantibodies to a p155/140 kDa doublet protein in juvenile dermatomyositis. Rheumatology [Internet]. 2007 Nov 28 [cited 2020 Jan 13];47(3):324–8. Available from: https://academic.oup.com/rheumatology/article-lookup/doi/10.1093/rheumatology/kem359

120. Sato JDO, Sallum AME, Ferriani VPL, Marini R, Sacchetti SB, Okuda EM, et al. A Brazilian registry of juvenile dermatomyositis: Onset features and classification of 189 cases. Clin Exp Rheumatol. 2009;27(6):1031–8.

121. So MW, Koo BS, Kim YG, Lee CK, Yoo B. Idiopathic inflammatory myopathy associated with malignancy: A retrospective cohort of 151 Korean patients with dermatomyositis and polymyositis. J Rheumatol. 2011 Nov;38(11):2432–5.

122. Antiochos BB, Brown LA, Li Z, Tosteson TD, Wortmann RL, Rigby WFC. Malignancy is associated with dermatomyositis but not polymyositis in Northern New England, USA. J Rheumatol. 2009 Dec;36(12):2704–10.

123. Stockton D, Doherty VR, Brewster DH. Risk of cancer in patients with dermatomyositis or polymyositis, and follow-up implications: a Scottish population-based cohort study. Br J Cancer [Internet]. 2001 Jul 6 [cited 2016 Jul 13];85(1):41–5. Available from: http://www.ncbi.nlm.nih.gov/pubmed/11437400

124. András C, Ponyi A, Constantin T, Csiki Z, Szekanecz É, Szodoray P, et al. Dermatomyositis and polymyositis associated with malignancy: A 21-year retrospective study. J Rheumatol. 2008 Mar;35(3):438–44.

125. Hill CL, Zhang Y, Sigurgeirsson B, Pukkala E, Mellemkjaer L, Airio A, et al. Frequency of specific cancer types in dermatomyositis and polymyositis: a population-based study. Lancet [Internet]. 2001 Jan 13 [cited 2018 Mar 13];357(9250):96–100. Available from: http://www.ncbi.nlm.nih.gov/pubmed/11197446

126. Wang J, Guo G, Chen G, Wu B, Lu L, Bao L. Meta-analysis of the association of dermatomyositis and polymyositis with cancer. Br J Dermatol [Internet]. 2013 Oct [cited 2020 Aug 7];169(4):838–47. Available from: https://pubmed.ncbi.nlm.nih.gov/23909921/

127. Neri R, Simone B, Iacopetti V, Iacopetti G, Pepe P, D’Ascanio A, et al. Cancer-associated myositis: A 35-year retrospective study of a monocentric cohort. Rheumatol Int. 2014;34(4):565–9.

128. Chen YJ, Wu CY, Shen JL. Predicting factors of malignancy in dermatomyositis and polymyositis: A case-control study. Br J Dermatol. 2001;144(4):825–31.

129. Marie I, Hatron PY, Levesque H, Hachulla E, Hellot MF, Michon-Pasturel U, et al. Influence of age on characteristics of polymyositis and dermatomyositis in adults. Medicine (Baltimore). 1999 May;78(3):139–47.

130. Lu X, Yang H, Shu X, Chen F, Zhang Y, Zhang S, et al. Factors Predicting Malignancy in Patients with Polymyositis and Dermatomyostis: A Systematic Review and Meta-Analysis. Kuwana M, editor. PLoS One [Internet]. 2014 Apr 8 [cited 2020 Jan 13];9(4):e94128. Available from: http://dx.plos.org/10.1371/journal.pone.0094128

131. Ponyi A, Constantin T, Garami M, András C, Tállai B, Váncsa A, et al. Cancer-associated myositis: clinical features and prognostic signs. Ann N Y Acad Sci [Internet]. 2005 Jun [cited 2016 Jun 23];1051:64–71. Available from: http://www.ncbi.nlm.nih.gov/pubmed/16126945

132. Azuma K, Yamada H, Ohkubo M, Yamasaki Y, Yamasaki M, Mizushima M, et al. Incidence and predictive factors for malignancies in 136 Japanese patients with dermatomyositis, polymyositis and clinically amyopathic dermatomyositis. Mod Rheumatol. 2011 Apr;21(2):178–83.

133. Basset-Seguin N, Roujeau JC, Gherardi R, Guillaume JC, Revuz J, Touraine R. Prognostic factors and predictive signs of malignancy in adult dermatomyositis. A study of 32 cases. Arch Dermatol [Internet]. 1990 May [cited 2020 Jan 13];126(5):633–7. Available from: http://www.ncbi.nlm.nih.gov/pubmed/2334184

134. Sparsa A, Liozon E, Herrmann F, Ly K, Lebrun V, Soria P, et al. Routine vs extensive malignancy search for adult dermatomyositis and polymyositis: A study of 40 patients. Arch Dermatol [Internet]. 2002;138(7):885–90. Available from: https://www.scopus.com/inward/record.uri?eid=2-s2.0-0036295692&partnerID=40&md5=49dedb70fd38b1ed7661a038484d93d0

135. Burnouf M, Mahe E, Verpillat P, Descamps V, Lebrun-Vignes B, Picard-Dahan C, et al. [Cutaneous necrosis is predictive of cancer in adult dermatomyositis]. Ann Dermatol Venereol. 2003 Mar;130(3):313–6.

136. Leow YH, Goh CL. Malignancy in adult dermatomyositis. Int J Dermatol. 1997;36(12):904–7.

137. Fardet L, Dupuy A, Gain M, Kettaneh A, Ch̃rin P, Bachelez H, et al. Factors associated with underlying malignancy in a retrospective cohort of 121 Patients with dermatomyositis. Medicine (Baltimore) [Internet]. 2009 Mar [cited 2020 Aug 12];88(2):91–7. Available from: https://pubmed.ncbi.nlm.nih.gov/19282699/

138. Targoff IN, Mamyrova G, Trieu EP, Perurena O, Koneru B, O’Hanlon TP, et al. A novel autoantibody to a 155-kd protein is associated with dermatomyositis. Arthritis Rheum. 2006 Nov;54(11):3682–9.

139. Kaji K, Fujimoto M, Hasegawa M, Kondo M, Saito Y, Komura K, et al. Identification of a novel autoantibody reactive with 155 and 140 kDa nuclear proteins in patients with dermatomyositis: An association with malignancy. Rheumatology. 2007 Jan;46(1):25–8.

140. Chinoy H, Fertig N, Oddis C V, Ollier WER, Cooper RG. The diagnostic utility of myositis autoantibody testing for predicting the risk of cancer-associated myositis. Ann Rheum Dis [Internet]. 2007 Oct 1 [cited 2017 Mar 24];66(10):1345–9. Available from: http://www.ncbi.nlm.nih.gov/pubmed/17392346

141. Fujikawa K, Kawakami A, Kaji K, Fujimoto M, Kawashiri S, Iwamoto N, et al. Association of distinct clinical subsets with myositis-specific autoantibodies towards anti-155/140-kDa polypeptides, anti-140-kDa polypeptides, and anti-aminoacyl tRNA synthetases in Japanese patients with dermatomyositis: a single-centre, cross-sectiona. Scand J Rheumatol [Internet]. 2009;38(4):263–7. Available from: http://www.ncbi.nlm.nih.gov/pubmed/19444719

142. Trallero-Araguás E, Labrador-Horrillo M, Selva-O’Callaghan A, Martínez MA, Martínez-Gómez X, Palou E, et al. Cancer-associated myositis and anti-p155 autoantibody in a series of 85 patients with idiopathic inflammatory myopathy. Medicine (Baltimore). 2010 Jan;89(1):47–52.

143. Bodoki L, Nagy-Vincze M, Griger Z, Betteridge Z, Szöllosi L, Dankó K. Four dermatomyositis-specific autoantibodies-anti-TIF1γ, anti-NXP2, anti-SAE and anti-MDA5-in adult and juvenile patients with idiopathic inflammatory myopathies in a Hungarian cohort. Vol. 13, Autoimmunity Reviews. Elsevier B.V.; 2014. p. 1211–9.

144. Ichimura Y, Matsushita T, Hamaguchi Y, Kaji K, Hasegawa M, Tanino Y, et al. Anti-NXP2 autoantibodies in adult patients with idiopathic inflammatory myopathies: possible association with malignancy. Ann Rheum Dis [Internet]. 2012;71(5):710–3. Available from: http://ovidsp.ovid.com/ovidweb.cgi?T=JS&PAGE=reference&D=med9&NEWS=N&AN=22258483

145. Fiorentino DF, Chung LS, Christopher-Stine L, Zaba L, Li S, Mammen AL, et al. Most patients with cancer-associated dermatomyositis have antibodies to nuclear matrix protein NXP-2 or transcription intermediary factor 1γ. Arthritis Rheum [Internet]. 2013 Nov [cited 2017 Feb 6];65(11):2954–62. Available from: http://www.ncbi.nlm.nih.gov/pubmed/24037894

146. Yang H, Peng Q, Yin L, Li S, Shi J, Zhang Y, et al. Identification of multiple cancer-associated myositis-specific autoantibodies in idiopathic inflammatory myopathies: A large longitudinal cohort study. Arthritis Res Ther. 2017;19(1).

147. Oldroyd A, Sergeant JC, New P, McHugh NJ, Betteridge Z, Lamb JA, et al. The temporal relationship between cancer and adult onset anti-transcriptional intermediary factor 1 antibody-positive dermatomyositis. Rheumatol (United Kingdom). 2019;58(4):650–5.

148. Zhong Z, Lin F, Yang J, Zhang F, Zeng X, You X. Pregnancy in polymyositis or dermatomyositis: Retrospective results from a tertiary centre in China. Rheumatol (United Kingdom). 2017;56(8):1272–5.

149. Váncsa A, Ponyi A, Constantin T, Zeher M, Dankó K. Pregnancy outcome in idiopathic inflammatory myopathy. Rheumatol Int. 2007;27(5):435–9.

150. Gutiéarrez G, Dagnino R, Mintz G. Polymyositis/dermatomyositis and pregnancy. Arthritis Rheum. 1984;27(3):291–4.

151. Kolstad KD, Fiorentino D, Li S, Chakravarty EF, Chung L. Pregnancy outcomes in adult patients with dermatomyositis and polymyositis. Semin Arthritis Rheum [Internet]. 2018 Jun 1 [cited 2021 Jul 2];47(6):865–9. Available from: https://pubmed.ncbi.nlm.nih.gov/29217291/

152. Iago PF, Albert SOC, Andreu FC, Xavier MG, Jose RP, Jordi PL, et al. ‘Pregnancy in adult-onset idiopathic inflammatory myopathy’: Report from a cohort of myositis patients from a single center. Semin Arthritis Rheum. 2014;44(2):234–40.

153. Limaye V, Hakendorf P, Woodman RJ, Blumbergs P, Roberts-Thomson P. Mortality and its predominant causes in a large cohort of patients with biopsy-determined inflammatory myositis. Intern Med J. 2012;42(2):191–8.

154. Linos E, Fiorentino D, Lingala B, Krishnan E, Chung L. Atherosclerotic cardiovascular disease and dermatomyositis: an analysis of the Nationwide Inpatient Sample survey. Arthritis Res Ther [Internet]. 2013;15:R7. Available from: http://www.ncbi.nlm.nih.gov/pubmed/23298514

155. Coyle K, Rother KI, Weise M, Ahmed A, Miller FW, Rider LG. Metabolic abnormalities and cardiovascular risk factors in children with myositis. J Pediatr [Internet]. 2009 [cited 2021 Jun 3];155(6):882–7. Available from: https://pubmed.ncbi.nlm.nih.gov/19643439/

156. Eimer MJ, Brickman WJ, Seshadri R, Ramsey-Goldman R, McPherson DD, Smulevitz B, et al. Clinical status and cardiovascular risk profile of adults with a history of juvenile dermatomyositis. J Pediatr [Internet]. 2011 Nov [cited 2021 Jun 3];159(5):795–801. Available from: https://pubmed.ncbi.nlm.nih.gov/21784434/

157. Silverberg JI, Kwa L, Kwa MC, Laumann AE, Ardalan K. Cardiovascular and cerebrovascular comorbidities of juvenile dermatomyositis in US children: An analysis of the National Inpatient Sample. Rheumatol (United Kingdom) [Internet]. 2018 Apr 1 [cited 2021 Jun 3];57(4):694–702. Available from: https://pubmed.ncbi.nlm.nih.gov/29373707/

158. DeVere R, Bradley WG. Polymyositis: its presentation, morbidity and mortality. Brain [Internet]. 1975 Dec [cited 2017 Mar 24];98(4):637–66. Available from: http://www.ncbi.nlm.nih.gov/pubmed/1218371

159. Denbow CE, Lie JT, Tancredi RG, Bunch TW. Cardiac involvement in polymyositis. Arthritis Rheum [Internet]. 1979 Oct [cited 2016 Jun 29];22(10):1088–92. Available from: http://doi.wiley.com/10.1002/art.1780221007

160. Zhang L, Wang G, Ma L, Zu N. Cardiac involvement in adult polymyositis or dermatomyositis: a systematic review. Clin Cardiol [Internet]. 2012 Nov [cited 2016 Jun 29];35(11):686–91. Available from: http://www.ncbi.nlm.nih.gov/pubmed/22847365

161. Dankó K, Ponyi A, Constantin T, Borgulya G, Szegedi G. Long-term survival of patients with idiopathic inflammatory myopathies according to clinical features: a longitudinal study of 162 cases. Medicine (Baltimore) [Internet]. 2004 Jan [cited 2016 Jun 29];83(1):35–42. Available from: http://www.ncbi.nlm.nih.gov/pubmed/14747766

162. Haupt HM, Hutchins GM. The heart and cardiac conduction system in polymyositis-dermatomyositis: A clinicopathologic study of 16 autopsied patients. Am J Cardiol [Internet]. 1982 Nov [cited 2016 Jun 29];50(5):998–1006. Available from: http://linkinghub.elsevier.com/retrieve/pii/0002914982904088

163. Kiely PDW, Bruckner FE. Serum skeletal troponin I in inflammatory muscle disease: Relation to creatine kinase, CKMB and cardiac troponin I [1] [Internet]. Vol. 59, Annals of the Rheumatic Diseases. Ann Rheum Dis; 2000 [cited 2021 Jul 2]. p. 750–1. Available from: https://pubmed.ncbi.nlm.nih.gov/11023449/

164. Allanore Y, Vignaux O, Arnaud L, Puéchal X, Pavy S, Duboc D, et al. Effects of corticosteroids and immunosuppressors on idiopathic inflammatory myopathy related myocarditis evaluated by magnetic resonance imaging. Ann Rheum Dis [Internet]. 2006 Feb [cited 2016 Jun 29];65(2):249–52. Available from: http://www.ncbi.nlm.nih.gov/pubmed/16410529

165. Aggarwal R, Lebiedz-Odrobina D, Sinha A, Manadan A, Case JP. Serum cardiac troponin T, but not troponin I, is elevated in idiopathic inflammatory myopathies. J Rheumatol [Internet]. 2009 Dec [cited 2021 Jul 2];36(12):2711–4. Available from: https://pubmed.ncbi.nlm.nih.gov/19833747/

166. Fisher C, Agrawal S, Wong WM, Fahie-Wilson M, Dasgupta B. Clinical observations on the significance of raised cardiac troponin-T in patients with myositis of varying etiologies seen in rheumatology practice. Clin Rheumatol [Internet]. 2010 [cited 2021 Jul 2];29(10):1107–11. Available from: https://pubmed.ncbi.nlm.nih.gov/20556454/

167. Rosenbohm A, Buckert D, Gerischer N, Walcher T, Kassubek J, Rottbauer W, et al. Early diagnosis of cardiac involvement in idiopathic inflammatory myopathy by cardiac magnetic resonance tomography. J Neurol [Internet]. 2015 Apr 12 [cited 2018 May 3];262(4):949–56. Available from: http://www.ncbi.nlm.nih.gov/pubmed/25673126

168. Diederichsen LP, Simonsen JA, Diederichsen AC, Hvidsten S, Hougaard M, Junker P, et al. Cardiac Abnormalities in Adult Patients With Polymyositis or Dermatomyositis as Assessed by Noninvasive Modalities. Arthritis Care Res [Internet]. 2016 Jul 1 [cited 2021 Jul 2];68(7):1012–20. Available from: https://pubmed.ncbi.nlm.nih.gov/26502301/

169. Lilleker JB, Diederichsen ACP, Jacobsen S, Guy M, Roberts ME, Sergeant JC, et al. Using serum troponins to screen for cardiac involvement and assess disease activity in the idiopathic inflammatory myopathies. Rheumatology [Internet]. 2018 Mar 12 [cited 2018 May 3]; Available from: https://academic.oup.com/rheumatology/advance-article/doi/10.1093/rheumatology/key031/4930651

170. Erlacher P, Lercher A, Falkensammer J, Nassonov EL, Samsonov MI, Shtutman VZ, et al. Cardiac troponin and β-type myosin heavy chain concentrations in patients with polymyositis or dermatomyositis. Clin Chim Acta [Internet]. 2001 [cited 2021 Jul 2];306(1–2):27–33. Available from: https://pubmed.ncbi.nlm.nih.gov/11282091/

171. Cantez S, Gross GJ, MacLusky I, Feldman BM. Cardiac findings in children with juvenile Dermatomyositis at disease presentation. Pediatr Rheumatol [Internet]. 2017 Jul 11 [cited 2021 Jul 2];15(1). Available from: https://pubmed.ncbi.nlm.nih.gov/28693511/

172. Schwartz T, Sanner H, Gjesdal O, Flatø B, Sjaastad I. In juvenile dermatomyositis, cardiac systolic dysfunction is present after long-term follow-up and is predicted by sustained early skin activity. Ann Rheum Dis [Internet]. 2014 [cited 2021 Jun 3];73(10):1805–10. Available from: https://pubmed.ncbi.nlm.nih.gov/23881732/

173. Barth Z, Witczak BN, Schwartz T, Gjesdal K, Flatø B, Koller A, et al. In juvenile dermatomyositis, heart rate variability is reduced, and associated with both cardiac dysfunction and markers of inflammation: A cross-sectional study median 13.5 years after symptom onset. Rheumatol (United Kingdom) [Internet]. 2016 Mar 1 [cited 2021 Jul 2];55(3):535–43. Available from: https://pubmed.ncbi.nlm.nih.gov/26500284/

174. Azola A, Mulheren R, Mckeon G, Lloyd T, Christopher-Stine L, Palmer J, et al. Dysphagia in Myositis: A Study of the Structural and Physiologic Changes Resulting in Disordered Swallowing. Am J Phys Med Rehabil [Internet]. 2020 May 1 [cited 2021 Jul 2];99(5):404–8. Available from: https://pubmed.ncbi.nlm.nih.gov/31764229/

175. Chiu SK, Yang YH, Wang LC, Chiang BL. Ten-year experience of juvenile dermatomyositis: A retrospective study. J Microbiol Immunol Infect [Internet]. 2007 Feb 1 [cited 2021 Jul 2];40(1):68–73. Available from: https://europepmc.org/article/med/17332910

176. Oh TH, Brumfield KA, Hoskin TL, Stolp KA, Murray JA, Basford JR. Dysphagia in inflammatory myopathy: Clinical characteristics, treatment strategies, and outcome in 62 patients. Mayo Clin Proc [Internet]. 2007 [cited 2021 Jul 2];82(4):441–7. Available from: https://pubmed.ncbi.nlm.nih.gov/17418072/

177. Casal-Dominguez M, Pinal-Fernandez I, Mego M, Accarino A, Jubany L, Azpiroz F, et al. High-resolution manometry in patients with idiopathic inflammatory myopathy: Elevated prevalence of esophageal involvement and differences according to autoantibody status and clinical subset. Muscle Nerve [Internet]. 2017 Sep [cited 2018 May 9];56(3):386–92. Available from: http://www.ncbi.nlm.nih.gov/pubmed/27935079

178. Williams RB, Grehan MJ, Hersch M, Andre J, Cook IJ. Biomechanics, diagnosis, and treatment outcome in inflammatory myopathy presenting as oropharyngeal dysphagia. Gut [Internet]. 2003 Apr 1 [cited 2021 May 12];52(4):471–8. Available from: www.gutjnl.com

179. Mugii N, Hasegawa M, Matsushita T, Hamaguchi Y, Oohata S, Okita H, et al. Oropharyngeal dysphagia in dermatomyositis: Associations with clinical and laboratory features including autoantibodies. PLoS One [Internet]. 2016 May 1 [cited 2020 Aug 12];11(5). Available from: https://pubmed.ncbi.nlm.nih.gov/27167831/

180. Mulcahy KP, Langdon PC, Mastaglia F. Dysphagia in inflammatory myopathy: Self-report, incidence, and prevalence. Dysphagia [Internet]. 2012 Mar [cited 2021 Jul 2];27(1):64–9. Available from: https://pubmed.ncbi.nlm.nih.gov/21442390/

181. Taieb A, Guichard C, Salamon R, Maleville J. Prognosis in Juvenile Dermatopolymyositis: A Cooperative Retrospective Study of 70 Cases. Pediatr Dermatol [Internet]. 1985 [cited 2021 Jul 2];2(4):275–81. Available from: https://pubmed.ncbi.nlm.nih.gov/4011505/

182. Marie I, Hachulla E, Hatron PY, Hellot MF, Levesque H, Devulder B, et al. Polymyositis and dermatomyositis: short term and longterm outcome, and predictive factors of prognosis. J Rheumatol [Internet]. 2001;28:2230–7. Available from: http://www.ncbi.nlm.nih.gov/pubmed/11669162

183. Sultan SM, Ioannou Y, Moss K, Isenberg DA. Outcome in patients with idiopathic inflammatory myositis: morbidity and mortality. Rheumatology [Internet]. 2002 Jan 1 [cited 2018 Nov 22];41(1):22–6. Available from: https://academic.oup.com/rheumatology/article-lookup/doi/10.1093/rheumatology/41.1.22

184. Langdon PC, Mulcahy K, Shepherd KL, Low VH, Mastaglia FL. Pharyngeal dysphagia in inflammatory muscle diseases resulting from impaired suprahyoid musculature. Dysphagia [Internet]. 2012 Sep [cited 2021 Jul 2];27(3):408–17. Available from: https://pubmed.ncbi.nlm.nih.gov/22207246/

185. Ertekin C, Seçil Y, Yüceyar N, Aydoǧdu I. Orophaxryngeal dysphagia in polymyositis/dermatomyositis. Clin Neurol Neurosurg [Internet]. 2004 Dec [cited 2021 Jul 2];107(1):32–7. Available from: https://pubmed.ncbi.nlm.nih.gov/15567550/

186. Kim SJ, Han TR, Jeong SJ, Beom JW. Comparison between swallowing-related and limb muscle involvement in dermatomyositis patients. Scand J Rheumatol [Internet]. 2010 Aug [cited 2021 Jul 2];39(4):336–40. Available from: https://www.tandfonline.com/doi/abs/10.3109/03009740903555366

187. McCann LJ, Garay SM, Ryan MM, Harris R, Riley P, Pilkington CA. Oropharyngeal dysphagia in juvenile dermatomyositis (JDM): An evaluation of videofluoroscopy swallow study (VFSS) changes in relation to clinical symptoms and objective muscle scores. Rheumatology [Internet]. 2007 Aug [cited 2021 Jul 2];46(8):1363–6. Available from: https://pubmed.ncbi.nlm.nih.gov/17569746/

188. Ogawa-Momohara M, Muro Y, Kono M, Akiyama M. Prognosis of dysphagia in dermatomyositis. Vol. 37, Clinical and Experimental Rheumatology. Clinical and Experimental Rheumatology S.A.S.; 2019. p. 165.

189. Marie I, Menard JF, Hatron PY, Hachulla E, Mouthon L, Tiev K, et al. Intravenous immunoglobulins for steroid-refractory esophageal involvement related to polymyositis and dermatomyositis: a series of 73 patients. Arthritis Care Res (Hoboken) [Internet]. 2010 [cited 2021 Jul 2];62(12):1748–55. Available from: https://pubmed.ncbi.nlm.nih.gov/20722047/

190. de Souza FHC, Levy-Neto M, Shinjo SK. Prevalence of clinical and laboratory manifestations and comorbidities in polymyositis according to gender. Rev Bras Reumatol [Internet]. 2011 [cited 2020 Jul 9];51(5):423–33. Available from: https://pubmed.ncbi.nlm.nih.gov/21952995/

191. Wang LY, Chen SF, Chiang JH, Hsu CY, Shen YC. Systemic autoimmune diseases are associated with an increased risk of obsessive–compulsive disorder: a nationwide population-based cohort study. Soc Psychiatry Psychiatr Epidemiol [Internet]. 2019;54(4):507–16. Available from: http://dx.doi.org/10.1007/s00127-018-1622-y

192. Wang LY, Chen SF, Chiang JH, Hsu CY, Shen YC. Autoimmune diseases are associated with an increased risk of schizophrenia: A nationwide population-based cohort study. Schizophr Res [Internet]. 2018;202:297–302. Available from: https://doi.org/10.1016/j.schres.2018.06.033

193. Armadans-Tremolosa I, Selva-O’Callaghan A, Visauta-Vinacua B, Guilera G, Pinal-Fernández I, Vilardell-Tarrés M. Health-related quality of life and well-being in adults with idiopathic inflammatory myopathy. Clin Rheumatol. 2014;33(8):1119–25.

194. Apaz MT, Saad-Magalhães C, Pistorio A, Ravelli A, de Oliveira sato J, Marcantoni MB, et al. Health-related quality of life of patients with juvenile dermatomyositis: Results from the paediatric rheumatology international trials organisation multinational quality of life cohort study. Arthritis Rheum [Internet]. 2009 Apr 15 [cited 2021 Jun 3];61(4):509–17. Available from: http://doi.wiley.com/10.1002/art.24343

195. Ponyi A, Borgulya G, Constantin T, Váncsa A, Gergely L, Dankó K. Functional outcome and quality of life in adult patients with idiopathic inflammatory myositis. Rheumatology [Internet]. 2005 Jan 1 [cited 2017 Sep 14];44(1):83–8. Available from: https://academic.oup.com/rheumatology/article-lookup/doi/10.1093/rheumatology/keh404

196. Tollisen A, Sanner H, Flatø B, Wahl AK. Quality of life in adults with juvenile-onset dermatomyositis: A case-control study. Arthritis Care Res. 2012;64(7):1020–7.

197. Poulsen KB, Alexanderson H, Dalgård C, Jacobsen S, Weile L, Diederichsen LP. Quality of life correlates with muscle strength in patients with dermato- or polymyositis. Clin Rheumatol. 2017;36(10):2289–95.

198. Marie I, Hatron PY, Cherin P, Hachulla E, Diot E, Vittecoq O, et al. Functional outcome and prognostic factors in anti-Jo1 patients with antisynthetase syndrome. Arthritis Res Ther [Internet]. 2013;15:R149. Available from: http://www.ncbi.nlm.nih.gov/pubmed/24286268

199. Goreshi R, Chock M, Foering K, Feng R, Okawa J, Rose M, et al. Quality of life in dermatomyositis. J Am Acad Dermatol [Internet]. 2011 Dec [cited 2017 Oct 19];65(6):1107–16. Available from: http://www.ncbi.nlm.nih.gov/pubmed/21722989

200. Hundley JL, Carroll CL, Lang W, Snively B, Yosipovitch G, Feldman SR, et al. Cutaneous symptoms of dermatomyositis significantly impact patients’ quality of life. J Am Acad Dermatol. 2006;54(2):217–20.

201. Shirani Z, Kucenic MJ, Carroll CL, Fleischer AB, Feldman SR, Yosipovitch G, et al. Pruritus in adult dermatomyositis. Clin Exp Dermatol. 2004;29(3):273–6.

202. Feldon M, Farhadi PN, Brunner HI, Itert L, Goldberg B, Faiq A, et al. Predictors of Reduced Health-Related Quality of Life in Adult Patients With Idiopathic Inflammatory Myopathies. Arthritis Care Res [Internet]. 2017 Nov 1 [cited 2021 Sep 17];69(11):1743–50. Available from: https://pubmed.ncbi.nlm.nih.gov/28118525/

203. Butbul Aviel Y, Stremler R, Benseler SM, Cameron B, Laxer RM, Ota S, et al. Sleep and fatigue and the relationship to pain, disease activity and quality of life in juvenile idiopathic arthritis and juvenile dermatomyositis. Rheumatology (Oxford). 2011;50(11):2051–60.

204. Challa D, Crowson CS, Niewold TB, Reed AM. Predictors of changes in disease activity among children with juvenile dermatomyositis enrolled in the Childhood Arthritis and Rheumatology Research Alliance (CARRA) Legacy Registry. Clin Rheumatol. 2018;37(4):1011–5.

205. Alexanderson H, Stenström CH, Jenner G, Lundberg I. The safety of a resistive home exercise program in patients with recent onset active polymyositis or dermatomyositis. Scand J Rheumatol [Internet]. 2000 [cited 2017 Oct 19];29(5):295–301. Available from: http://www.ncbi.nlm.nih.gov/pubmed/11093595

206. Regardt M, Welin Henriksson E, Alexanderson H, Lundberg IE. Patients with polymyositis or dermatomyositis have reduced grip force and health-related quality of life in comparison with reference values: an observational study. Rheumatology [Internet]. 2011 Mar 1 [cited 2017 Oct 19];50(3):578–85. Available from: http://www.ncbi.nlm.nih.gov/pubmed/21097879

207. Koh ET, Seow A, Ong B, Ratnagopal P, Tjia H, Chng HH. Adult onset polymyositis/dermatomyositis: clinical and laboratory features and treatment response in 75 patients. Ann Rheum Dis [Internet]. 1993;52:857–61. Available from: http://www.ncbi.nlm.nih.gov/pubmed/8311535

208. Pinal-Fernandez I, Casal-Dominguez M, Huapaya JA, Albayda J, Paik JJ, Johnson C, et al. A longitudinal cohort study of the anti-synthetase syndrome: Increased severity of interstitial lung disease in black patients and patients with anti-PL7 and anti-PL12 autoantibodies. Rheumatol (United Kingdom) [Internet]. 2017 Jun 1 [cited 2020 Sep 1];56(6):999–1007. Available from: https://pubmed.ncbi.nlm.nih.gov/28339994/

209. Narang NS, Casciola-Rosen L, Li S, Chung L, Fiorentino DF. Cutaneous ulceration in dermatomyositis: Association with anti-melanoma differentiation-associated gene 5 antibodies and interstitial lung disease. Arthritis Care Res [Internet]. 2015 May 1 [cited 2021 Jul 2];67(5):667–72. Available from: https://pubmed.ncbi.nlm.nih.gov/25331610/

210. Satoh M, Krzyszczak ME, Li Y, Ceribelli A, Ross SJ, Chan EKL, et al. Frequent coexistence of anti-topoisomerase I and anti-U1RNP autoantibodies in African American patients associated with mild skin involvement: A retrospective clinical study. Arthritis Res Ther [Internet]. 2011 May 10 [cited 2021 Jul 2];13(3). Available from: https://pubmed.ncbi.nlm.nih.gov/21569292/

211. O’Hanlon TP, Rider LG, Schiffenbauer A, Targoff IN, Malley K, Pandey JP, et al. Immunoglobulin gene polymorphisms are susceptibility factors in clinical and autoantibody subgroups of the idiopathic inflammatory myopathies. Arthritis Rheum [Internet]. 2008 Oct [cited 2021 Jul 2];58(10):3239–46. Available from: https://pubmed.ncbi.nlm.nih.gov/18821675/

212. O’Hanlon TP, Rider LG, Mamyrova G, Targoff IN, Arnett FC, Reveille JD, et al. HLA polymorphisms in African Americans with idiopathic inflammatory myopathy: Allelic profiles distinguish patients with different clinical phenotypes and myositis autoantibodies. Arthritis Rheum [Internet]. 2006 Nov 1 [cited 2021 Jul 2];54(11):3670–81. Available from: https://onlinelibrary.wiley.com/doi/10.1002/art.22205

213. Reed AM, Stirling JD. Association of the HLA-DQA1*0501 allele in multiple racial groups with juvenile dermatomyositis. Hum Immunol [Internet]. 1995 [cited 2021 Jul 2];44(3):131–5. Available from: https://pubmed.ncbi.nlm.nih.gov/8666549/
