# Supplementary material for: British Society for Rheumatology guideline on management of paediatric, adolescent and adult patients with idiopathic inflammatory myopathy
Source: Rheumatology (Oxford). 2022 Mar 31;61(5):1760–8. doi: 10.1093/rheumatology/keac115 (PMC9398208; doi:10.1093/rheumatology/keac115)
Supplement: keac115_Supplementary_Data [file keac115_supplementary_data.zip › keac115-suppl_data/Suppl_table_S2_-_Audit_tool_IIM_-_Final_FL.docx]

**Supplementary Table 2 - Audit tool IIM**

Scope:

The purpose of this audit tool is to ensure that the BSR IIM guideline (2021) is being adhered to. This audit should be undertaken on a sample of patients attending secondary / tertiary care.

|  |  | YES / NO (✓ or ✗) | N/A (✓) | |
| --- | --- | --- | --- | --- |
| 1 | In the presence of muscle inflammation, was the patient treated with high dose corticosteroid |  | No muscle inflammation |  |
| 2 | Was intravenous methylprednisolone given / considered in the presence of concerns about gastrointestinal absorption |  | No concern with gastro-intestinal absorption |  |
| 3 | Was corticosteroid tapered as the patient responded to treatment |  | Not yet able to taper |  |
| 4 | In the presence of muscle inflammation, was the patient treated with a disease modifying anti-rheumatic drug |  | No muscle inflammation |  |
| 5 | For juvenile onset disease, was the combination of corticosteroid and methotrexate used as first line treatment |  | Adult onset or severe disease requiring alternative therapy |  |
| 6 | In the presence of severe / refractory muscle inflammation was treatment escalated with the use of IVIG, csDMARD, cyclophosphamide, rituximab, or other biologic therapy |  | No evidence of severe / refractory disease |  |
| 7 | In the presence of active muscle disease, did management include a safe and appropriate exercise programme led and monitored by a physiotherapist and /or occupational therapist? |  | No muscle inflammation |  |
| 8 | In the presence of active skin disease, did the patient receive treatment with csDMARD, IVIG or rituximab? |  | No active skin disease |  |
| 9 | Was sun avoidance and the use of high factor sunblock considered / advised |  | No active skin disease |  |
| 10 | In adults, was screening carried out for Interstitial Lung Disease (ILD) |  |  |  |
| 11 | In the presence of ILD in adult onset IIM, was treatment given with high dose corticosteroid, csDMARD, cyclophosphamide or rituximab? |  | No evidence of ILD |  |
| 12 | In adults with IIM, was a bone health assessment carried out? |  | Juvenile onset disease |  |
| 13 | For juvenile onset IIM, was care provided by paediatric specialists? |  | Adult onset disease |  |
| 14 | Were age specific considerations taken into account when using tools measuring muscle strength, function and quality of life? |  | Age specific considerations do not apply for this patient |  |
| 15 | Were signs of connective tissue overlap looked for in those with juvenile onset IIM |  | Adult onset disease |  |
| 16 | Was an assessment carried out for calcinosis in those with juvenile onset IIM |  | Adult onset disease |  |
| 17 | Was the patient tested for myositis specific antibodies |  |  |  |
| 18 | For adult onset disease, was risk of cancer considered and appropriate screening carried out |  | Juvenile onset disease |  |
| 19 | For pregnant patients, was pregnancy managed in conjunction with maternal medicine specialists with on-going vigilance post-partum |  | No pregnancy |  |
| 20 | Did the patient undergo screening for cardiac involvement |  |  |  |
| 21 | Was the patient assessed and managed for cardiovascular risk factors |  | Juvenile onset & not thought to be at risk |  |
| 22 | Was the patient assessed for dysphagia |  |  |  |
| 23 | Was psychological well-being and health related quality of life considered / assessed |  |  |  |
| 24 | Was exercise / rehabilitation encouraged |  |  |  |
| 25 | Were targeted exercises given by a physiotherapist or occupational therapist to help activities of daily living |  | No difficulty with activities of daily living |  |
| 26 | Was ethnicity taken into account when considering risk factors for disease / comorbidities |  | No additional risk for ethnic / racial group |  |
