# Supplementary figures and images for: British Society for Rheumatology guideline on management of paediatric, adolescent and adult patients with idiopathic inflammatory myopathy
Source: Rheumatology (Oxford). 2022 Mar 31;61(5):1760–8. doi: 10.1093/rheumatology/keac115 (PMC9398208; doi:10.1093/rheumatology/keac115)

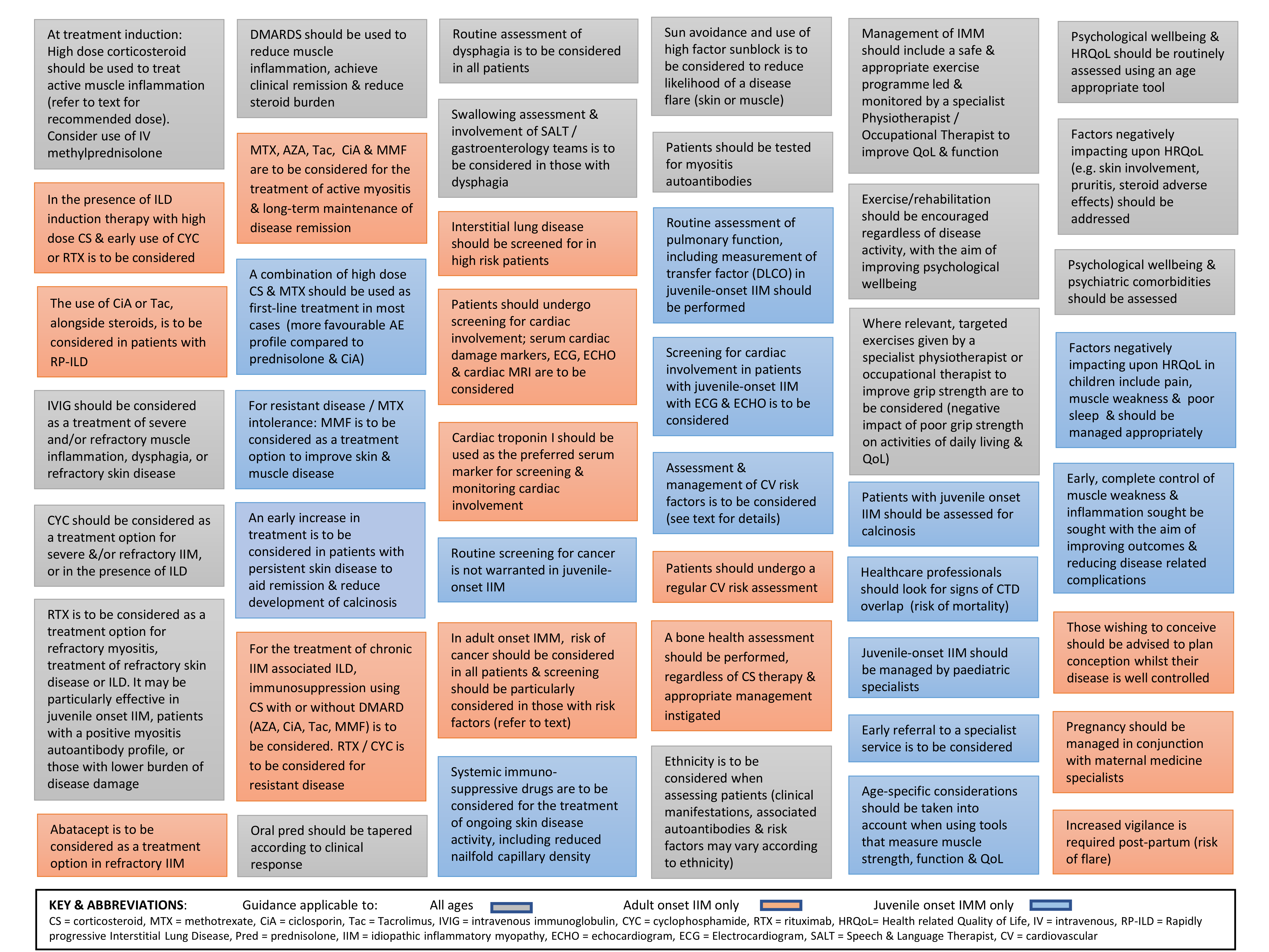

Supplement: keac115_Supplementary_Data [file keac115_supplementary_data.zip › keac115-suppl_data/Suppl_fig_S2_-_Image_of_all_recommendations_-_Final.tif]
